# Microenvironment-triggered dual-activation of a photosensitizer- fluorophore conjugate for tumor specific imaging and photodynamic therapy

**DOI:** 10.1038/s41598-020-68847-w

**Published:** 2020-07-22

**Authors:** Chang Wang, Shengdan Wang, Yuan Wang, Honghai Wu, Kun Bao, Rong Sheng, Xin Li

**Affiliations:** 1 College of Pharmaceutical Sciences, Zhejiang University, Hangzhou, 310058 China; 2 Collaborative Innovation Centre of Yangtze River Delta Region Green Pharmaceuticals, Zhejiang University of Technology, Hangzhou, 310014 China

**Keywords:** Chemical biology, Chemical modification

## Abstract

Photodynamic therapy is attracting increasing attention, but how to increase its tumor-specificity remains a daunting challenge. Herein we report a theranostic probe **(azo-PDT)** that integrates pyropheophorbide α as a photosensitizer and a NIR fluorophore for tumor imaging. The two functionalities are linked with a hypoxic-sensitive azo group. Under normal conditions, both the phototoxicity of the photosensitizer and the fluorescence of the fluorophore are inhibited. While under hypoxic condition, the reductive cleavage of the azo group will restore both functions, leading to tumor specific fluorescence imaging and phototoxicity. The results showed that **azo-PDT** selectively images BEL-7402 cells under hypoxia, and simultaneously inhibits BEL-7402 cell proliferation after near-infrared irradiation under hypoxia, while little effect on BEL-7402 cell viability was observed under normoxia. These results confirm the feasibility of our design strategy to improve the tumor-targeting ability of photodynamic therapy, and presents **azo-PDT** probe as a promising dual functional agent.

## Introduction

**Figure 1:**
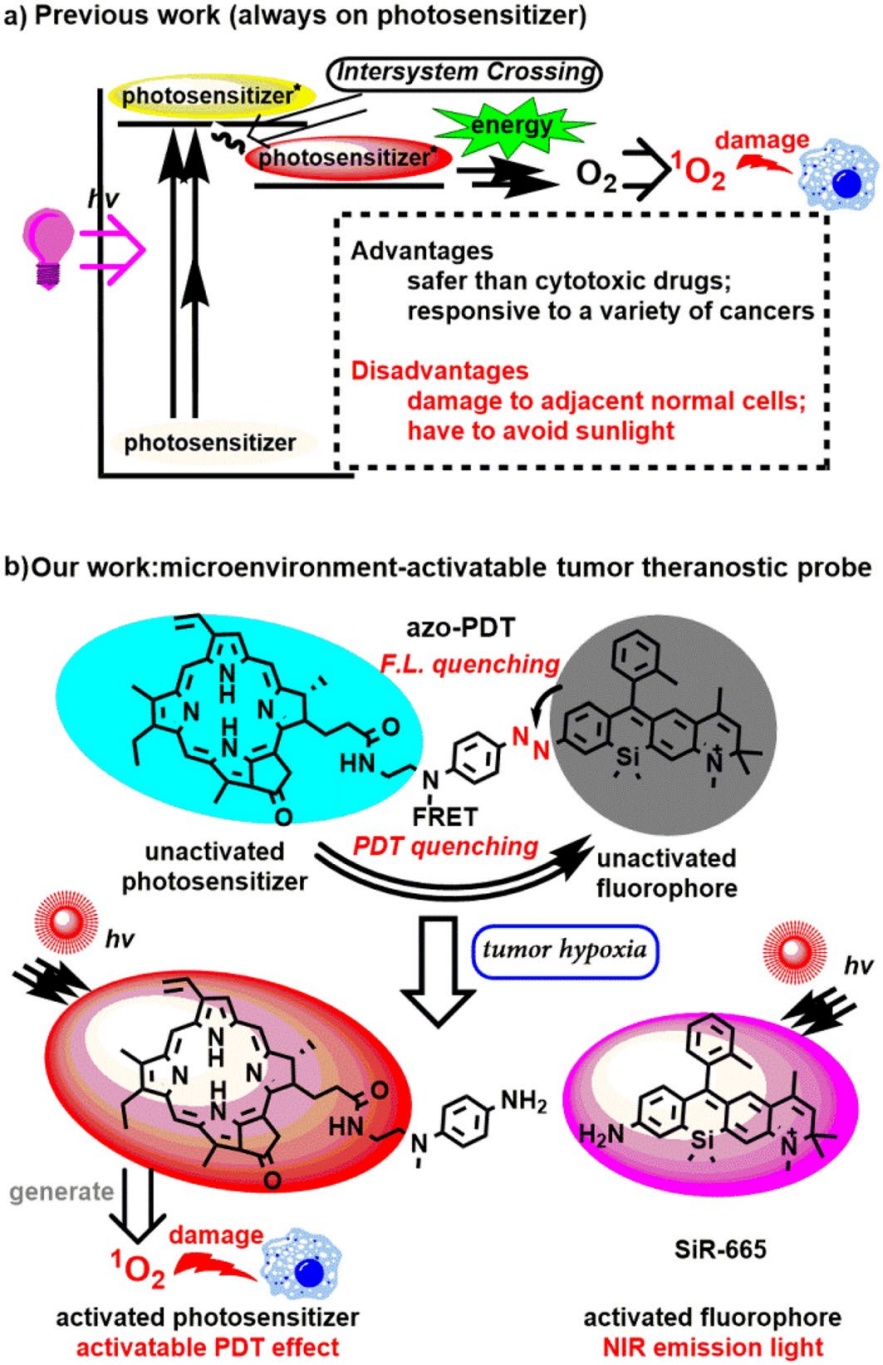
**(a)** Brief mechanism for ^1^O_2_ generation in photodynamic therapy; **(b)** brief activation mechanism of **azo-PDT** under tumour hypoxia situations.

Cancer is one of the most common causes of death, and more and more therapeutic strategies against this fatal disease have emerged in the past few decades. Among these strategies, photodynamic therapy has attracted much attention^1^. This therapy is based on singlet oxygen produced by photosensitizers under the irradiation with light of a specific wavelength to damage tumor tissues (Fig. 1a). Since the photo-damaging effect is induced by the interaction between a photosensitizer and light, tumor-specific therapy may be realized by focusing the light to the tumor site. Therefore, this therapeutic strategy is supposed to harm healthy tissue less than traditional cytotoxic drugs. Photofrin, the first photodynamic therapy drug approved by the FDA, has been routinely used for the treatment of certain cancers, such as esophageal cancer, lung cancer, bladder cancer, cervical cancer, and skin cancer^2^. However, since Photofrin is always ready to undergo photochemical reactions to produce singlet oxygen in the presence of light (630 nm) and also exhibits a long tissue retention time, it may cause long-lasting cutaneous photosensitivity^3^. Therefore, patients who have been treated with Photofrin have to avoid sunlight for several weeks, which presents a universal limit for “always on” photosensitizers.

To improve the tumor specificity, two strategies are generally utilized^1^. The first one is the conjugation of photosensitizers to nanocarriers with tumor-targeted, controlled-release properties. While this strategy has shown promise in preclinical models, it is limited by a complicated formulation process. The second strategy is based on prodrug-like photosensitizers whose photoreactivity can only be activated in tumor-specific environments. Tumor tissues usually demonstrate typical microenvironments that are significantly different from those of normal tissues. The features of these tumor microenvironments have been widely used for the design of tumor-targeted therapy^4,5^. Hypoxia is an important microenvironmental factor in cancer^6^. Therapies that are originally inert but sensitive to hypoxia may provide tumor-specificity. Taking advantage of hypoxia in solid tumors, many efforts have been devoted to develop tumor-targeted therapeutic or imaging agents^7–16^. Nagano and Urano et al. first reported that the azo group is sensitive toward hypoxia and may be employed to design fluorogenic probes for the detection of hypoxia^17^. Furthermore, they used the azo group as a quencher to design hypoxia-activated photosensitizers^12^. Recently, the groups of Tan, Fang, and Zhao collaborated to successfully design a hypoxia-activated aptamer for cancer imaging with improved specificity^13^. Based on hypoxia, Pu et al. successfully activated a prodrug of the chemotherapeutic drug Br-IPM^14^. All these results prove the advantage of the hypoxic tumor environment as a target to design tumor-specific imaging or therapy modalities. While many studies have been reported on hypoxia-activated therapeutic or imaging agents, hypoxia-dependent dual activation for simultaneous tumor imaging and photodynamic therapy has not been investigated to date, to the best of our knowledge.

To design bifunctional probes for hypoxia-dependent tumor imaging and photodynamic therapy, we started by interrogating the mechanism by which photosensitizers convert light energy into singlet oxygen. When a chromophore absorbs a photon, it is promoted to an excited state. The excited chromophore can lose energy by populating the first excited singlet state via internal conversion followed by rapid relaxation back to the ground state. However, for a photosensitizer, the excited singlet state electron is easily undergoing spin inversion to populate the first excited triplet state at lower energies via intersystem crossing. This triplet state readily interacts with ground-state molecular oxygen (^3^O_2_), which is a triplet state, leading to the production of radicals and reactive oxygen species^1^. Obviously, photochemical processes catalyzed by photosensitizers rely on the population of their triplet state. We hypothesized that inhibition of this state with a hypoxia-sensitive trigger may block the photochemical reaction under normoxia. Under hypoxia, the hypoxia-sensitive trigger switches on the photosensitizers and makes them ready for the photochemical reaction, leading to a hypoxia-specific photodynamic effect. Fluorescence resonance energy transfer (FRET) is the absorption of the energy of a donor in its excited state by an acceptor structure, which may be employed to absorb the energy of an excited photosensitizer to inhibit the population in its excited triplet state. Herein, utilizing the FRET mechanism, we conjugated a photosensitizer (as energy donor) and a near-infrared fluorophore (as energy acceptor) with an azo group as hypoxia trigger, to successfully design a new chemical entity (**azo-PDT**) that can not only detect solid tumors based on its hypoxia-activated fluorogenic response but also show hypoxia-specific phototoxicity for the ablation of tumor cells.

## Results and discussion

### Design and synthesis

We chose pyropheophorbide α (**Pyro**), which is an analog of **Photofrin**, as the photosensitizer for our proof-of-concept study due to its easy availability. For efficient FRET, donor emission and acceptor absorption should be adjacent and overlap^18^. Given the extremely low emission of **Pyro** around 700–800 nm, we reasoned that the Si-rhodamine fluorescent dye **SiR-665** could act as the energy acceptor due to its absorption around 600–750 nm^19^. Another advantage of **SiR-665** is its strong NIR II emission, which qualifies it as a fluorophore for tumor imaging. We designed the entity **azo-PDT**, which uses the azo group as hypoxia-specific trigger and also as linker between the photosensitizer **Pyro** and the fluorophore **SiR-665**. We reasoned that due to the FRET effect between **Pyro** and **SiR-665**, no photodynamic effect should be observed, not even under irradiation. Due to the azo-caused fluorescence quenching of **SiR-665**^17^, the probe should be non-emissive. Hypoxia will lead to the reductive cleavage of the azo group^17^, effectively separating the two functional moieties and, thus, activating both activity (Fig. 1b).

**Figure 2:**
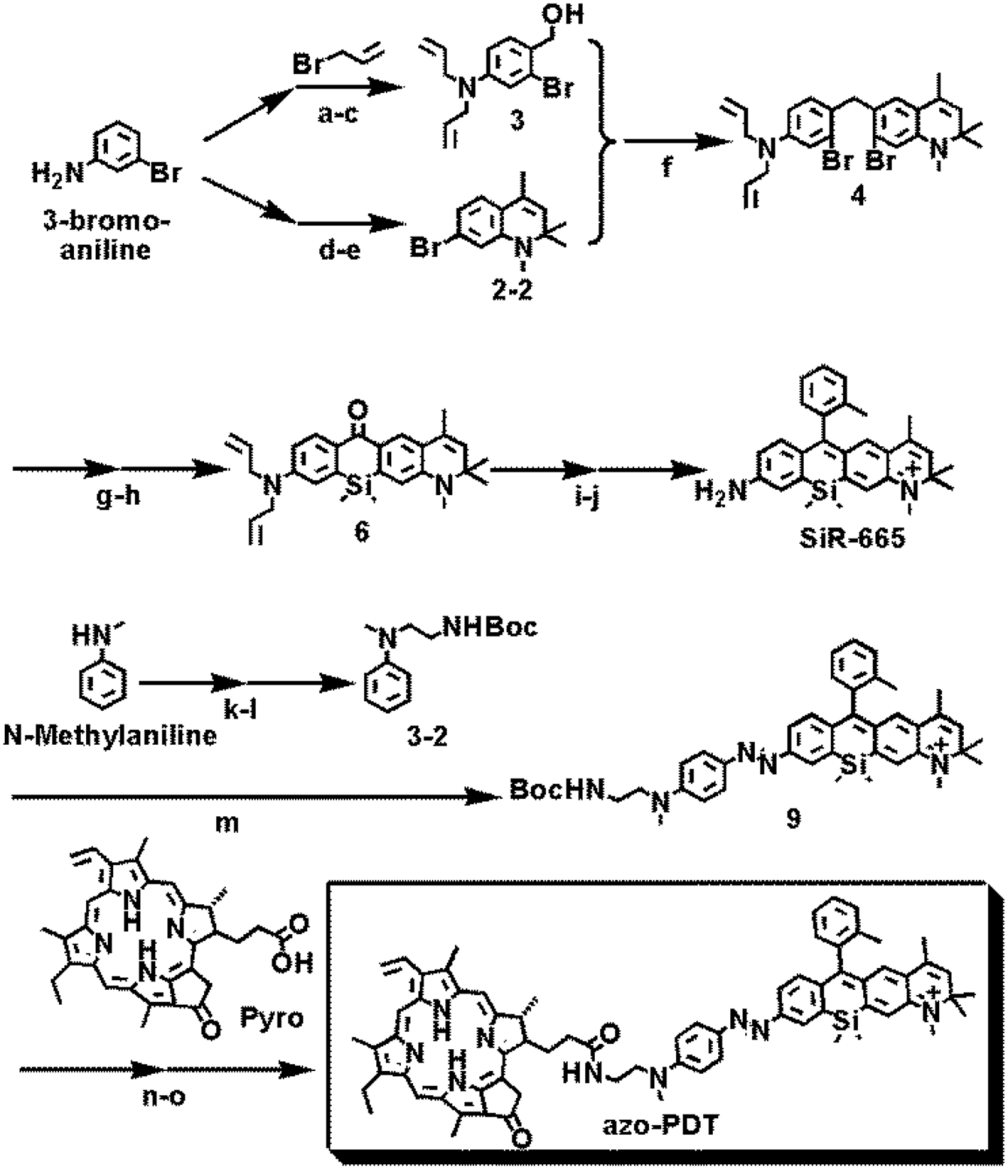
Synthesis of **azo-PDT**. Detailed reaction procedures were described in Scheme S1.

Procedures for the synthesis of **azo-PDT** are outlined in Fig. 2 and detailed in Scheme S1. Briefly, starting with 3-bromoaniline, we obtained **comp.3** and **comp.2–2**. Friedel–Crafts reaction between **comp.3** and **comp.2–2** yielded the intermediate **comp.4**, which was further converted into **SiR–665** following a reported procedures^19^. Diazo reaction of **SiR-665** with **comp.3–2** gave the azobenzene intermediate **comp.9**, and coupling of **comp.9** with **Pyro** yielded the desired compound **azo-PDT** (Fig. 2). The structures of **azo-PDT** and all precursors were characterized by NMR and mass spectrometry.

### Characterization of the photophysical properties of azo-PDT

**Figure 3:**
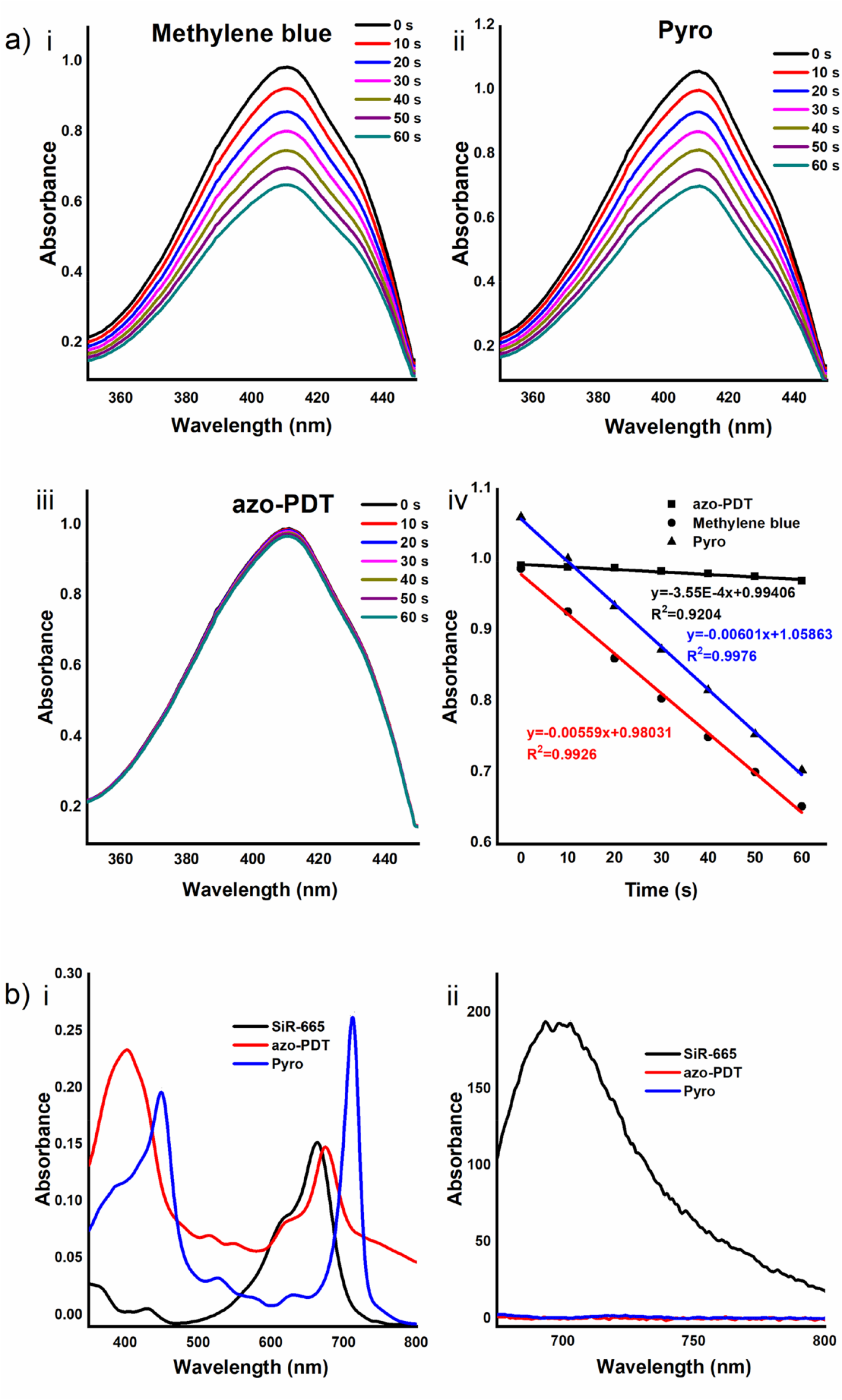
**(a)** (i–iii) Change of DPBF absorption spectra after photo-irradiation of the photosensitizers. Methylene blue (i), **Pyro** (ii) or **azo-PDT** (iii) was dissolved at 1 μM in EtOH. DPBF was added to the solution to a final concentration of 40 μM. The solution was irradiated with light filtered around the maximum absorption wavelength (670 nm). The absorption spectrum was obtained every 10 s. (iv) Decay curves of the 410 nm absorption bands in i, ii, and iii. The power of LED lamp was 150 W and the distance of irradiation was 80 cm; **(b)** Absorption (i) and fluorescent (ii) spectra of 5 µM of **SiR-665**, **azo-PDT** and **Pyro** in PBS.

We studied the photophysical properties of **azo-PDT** by first testing if its photoreactivity and fluorescence were quenched. For this purpose, we first measured the absorption and emission properties of **azo-PDT** in comparison with those of **SiR–665** (Table 1). Then, the singlet oxygen quantum yield (Φ(^1^O_2_)) of **azo-PDT** was quantitatively measured in comparison with that of **Pyro** according to a reported method^20^. 1,3-Diphenylisobenzofuran (DPBF) was used as the ^1^O_2_-capture and detection agent, and an EtOH solution of **azo-PDT** or **Pyro** in the presence of DPBF was irradiated at 670 nm. Once ^1^O_2_ is photo-induced, it would react with DPBF via 1,4-cycloaddition, resulting in a decrease of its absorbance at 410 nm (Fig. 3a). Using methylene blue (Φ(^1^O_2_) = 0.49) as a standard^21^, the Φ(^1^O_2_) values of **azo-PDT** and **Pyro** were calculated as 0.04 and 0.63, respectively (Table 1). The significantly lower Φ(^1^O_2_) value of **azo-PDT** compared with **Pyro** suggests effective quenching of the photoreactivity of **azo-PDT** due to the efficient FRET process between **Pyro** and **SiR-665**. This is in accord with the photophysical data, which showed partial overlap between the emission of **Pyro** and the absorption of **SiR-665** (Fig. 3b).

**Table 1:** Photophysical and photochemical parameters of **azo-PDT**, methylene blue, **SiR-665** and **pyro**.

Compound	λ_abs_ (nm)	λ_ex_ (nm)	λ_em_ (nm)	Φ_Δ_^c^
**Azo-PDT**	674^a^, 668^b^	/	/	0.04^b^
Methylene blue	654^b^	–	–	0.49^b^
**SiR-665**	664^a^	665^a^	693^a^	–
**Pyro**	667^b^	/	/	0.63^b^

^a^The data were obtained in PBS.

^b^The data were measured in EtOH.

^c^Φ_Δ_: singlet oxygen quantum yield. For methylene blue, Φ_Δ_ = 0.49. “/” means not detectable, and “–” means not detected.

After confirming the effective quenching of the ^**1**^**O**_**2**_**-**production ability of **azo-PDT**, we measured its fluorescence properties. As shown in Fig. 3b, **azo-PDT** is almost non-fluorescent, although SiR-665 is highly emissive. This finding indicates that the fluorescence of **SiR-665** fluorophore is significantly quenched by the azo group.

### Hypoxia-activated fluorescence of azo-PDT in aqueous solution

**Figure 4:**
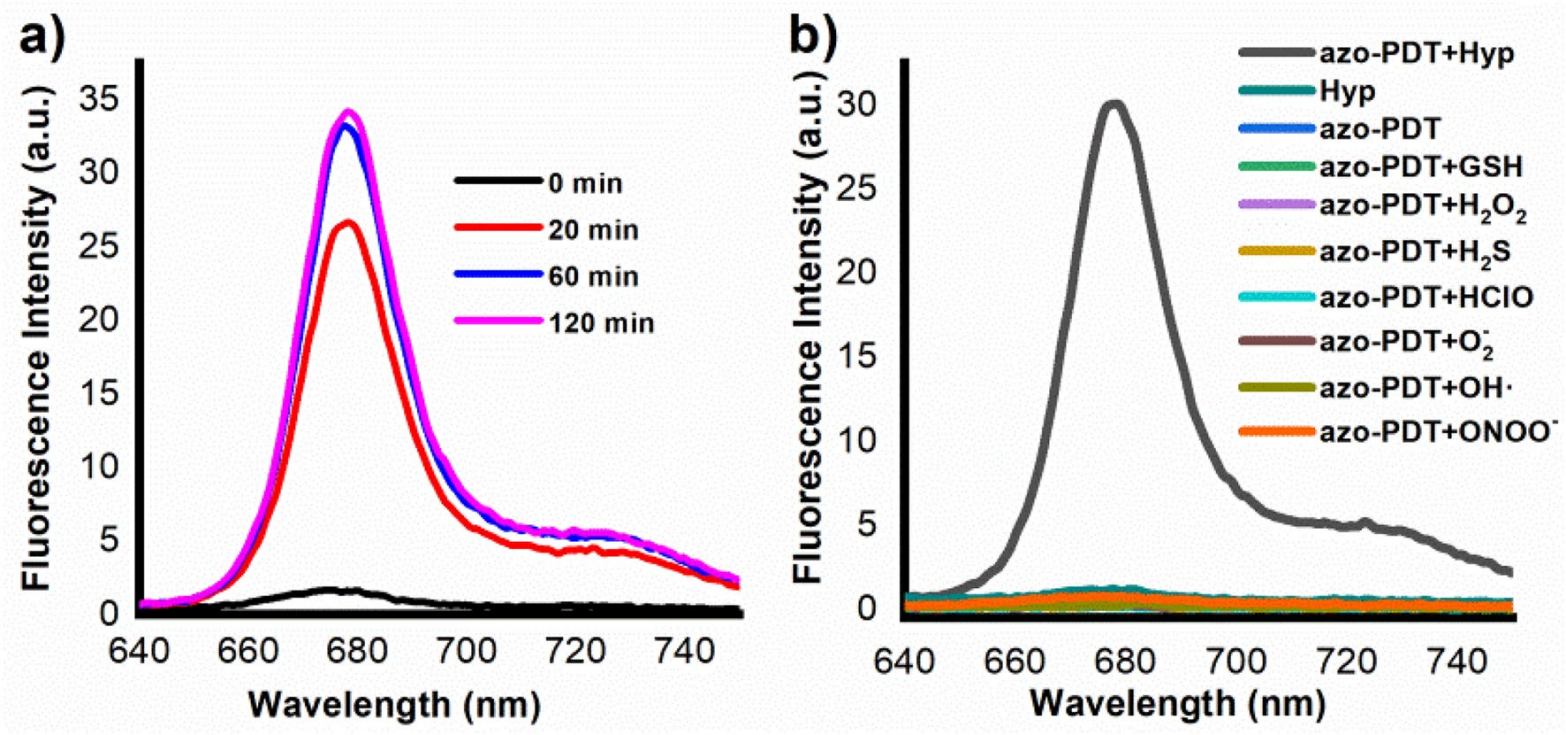
**(a)** Time-dependent changes in the fluorescence intensity of **azo-PDT** (5 μM) in the presence of mouse liver microsomes (226 μg/3 mL); **(b)** fluorescent responses of **azo-PDT** (5 μM) towards various analytes. Data shown were the fluorescence intensity of the probe at 680 nm after being incubated with various analytes for 60 min. All fluorescence data were collected in PBS (pH 7.4, 100 mM) containing 5% DMF as co-solvent at 37 °C with λ_ex_ 420 nm.

After confirming the effective inhibition of the ^1^O_2_-generation ability of **azo-PDT** and quenching of its fluorescence emission, we tested if reductive cleavage of the azo group restores both activities. For this purpose, the biological reductive hypoxic environment was mimicked with mouse liver microsomes^17^. After treating **azo-PDT** (5 μM) with mouse liver microsomes (75 μg/mL) in the presence of NADPH (50 μM) as a reductase cofactor, an increase of the probe fluorescence was observed, which intensified with the proceeding of the incubation (Fig. 4a).

We also checked if other biological species commonly found in live cells induce the switch-on of the fluorescence of **azo-PDT**. For this purpose, **azo-PDT** in PBS was treated with various analytes for 1 h, and then its fluorescence was observed. As shown in Fig. 4b, microsomes were the only analytes to induce its fluorescence, suggesting that **azo-PDT** is specifically activated by the biological hypoxic environment. This is highly advantageous, as this suggests that **azo-PDT** should have a tumor-specific photodynamic effect.

Furthermore, we also confirmed the reductive cleavage of the azo bond in **azo-PDT** to yield the free amino-**SiR-665** by LC–MS analysis (Fig. S1), supporting our design rationale.

### Hypoxia-activated fluorescence and photoreactivity of azo-PDT in live cells

Having confirmed the hypoxia-activated fluorescence of **azo-PDT** in aqueous solution, we studied if **azo-PDT** retains this feature in live cells for hypoxia-specific imaging and photodynamic ablation. For this purpose, we first screened a panel of cells to select the most sensitive one and applied **azo-PDT** to these cells under hypoxia (1% O_2_). All cells demonstrated higher intracellular probe fluorescence under hypoxia than under normoxia (Fig. S2), suggesting that **azo-PDT** may be activated under hypoxia in all tested cell lines. It is noteworthy that BEL-7402 cells showed the most dramatic intracellular fluorescence increase under hypoxia, suggesting that **azo-PDT** is more sensitive in BEL-7402 cells, which were thus subject of further research.

**Figure 5:**
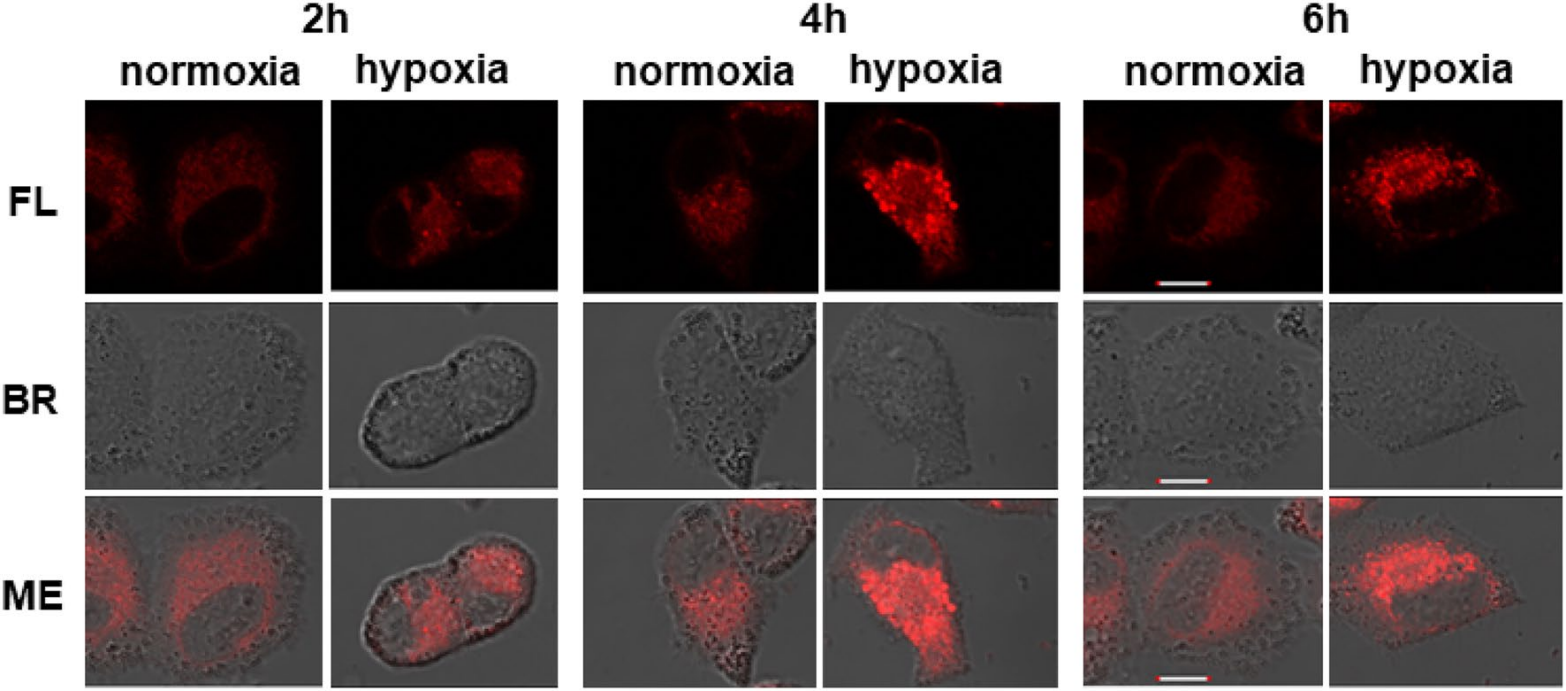
Time-dependent change of the fluorescence intensity from BEL-7402 cells with 5 μM **azo-PDT** containing DMSO (0.1%) and RH40 (0.1%) as cosolvent. Scale bar: 10 μm. *FL* fluorescence image, *BR* bright field image. The excitation and emission wavelengths were 640 nm and 650–750 nm, respectively.

First, the influence of the hypoxia incubation time on the activation of **azo-PDT** was studied by detecting the fluorescence switch-on effect. For this purpose, BEL-7402 cells were incubated with **azo-PDT** (5 μM) under hypoxia or normoxia for various times. While no significant time-dependent increase of the intracellular fluorescence was observed in the normoxia group, the intracellular **azo-PDT** fluorescence increased with increasing incubation time in the hypoxia group. These results suggest that, under hypoxia, the azo group in **azo-PDT** is reductively cleaved, which occurs gradually with increasing incubation times (Fig. 5).

In the following, we optimized the working concentration of **azo-PDT** to stain cells. For this purpose, BEL-7402 cells were incubated with various concentrations of **azo-PDT** under normoxia or hypoxia for 6 h. Cells under normoxia demonstrated negligible intracellular **azo-PDT** fluorescence, indicating that the fluorescence of **azo-PDT** is quenched, while the intracellular fluorescence of cells under hypoxia depended on the **azo-PDT** concentration (Fig. S3). Our results showed that an **azo-PDT** concentration of 2.5 μM was sufficient to yield significant intracellular fluorescence under hypoxia. Therefore, we chose this **azo-PDT** working concentration and an incubation time of 6 h for the following cell experiments. Under these conditions, the intracellular fluorescence intensity of **azo-PDT** was 1.8-fold higher under hypoxia than under normoxia (Fig. S4).

**Figure 6:**
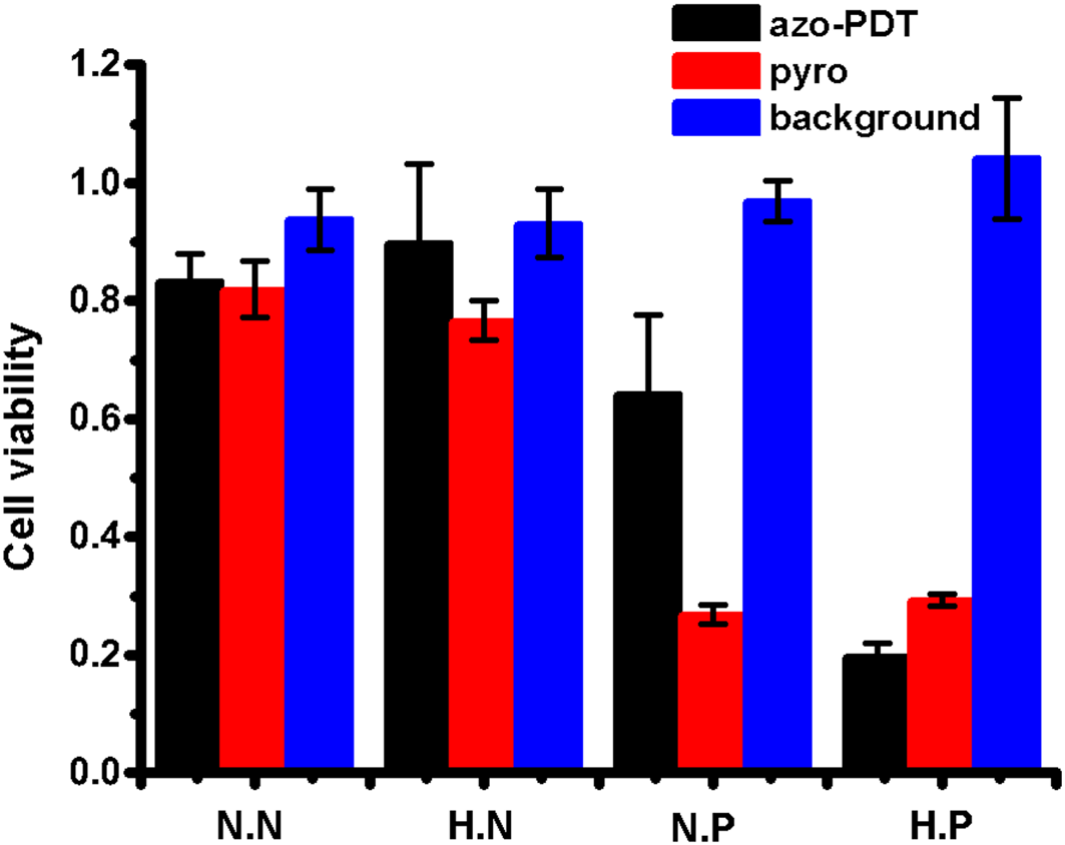
Survival viabilities of BEL-7402 cells after the treatment of **azo-PDT** or **Pyro** at 2.5 μM under normoxia or hypoxia, followed with or without photoirradiation. SRB assay was used to monitor cell viabilities. *N.N* normoxia with no photoirradiation, *H.N* hypoxia with no photoirradiation, *N.P* normoxia with photoirradiation, *H.P* hypoxia with photoirradiation. The power of LED lamp was 150 W and the distance of irradiation was 6.5 cm.

After confirming the hypoxia-dependent activation of the fluorescence of **azo-PDT** in BEL-7402 cells, we tested if hypoxia also restores the ^1^O_2_-generation ability of **azo-PDT** in BEL-7402 cells. BEL-7402 cells were incubated with **azo-PDT** under normoxia or hypoxia for 6 h and then irradiated with LED light at 670 nm for 20 min to induce the production of ^1^O_2_. Then, the cells were incubated without irradiation for another 24 h, followed by SRB assay and cck–8 assay to measure the cell viability using **Pyro** as a positive control. While the **Pyro** group showed an irradiation-dependent cell ablation effect under both normoxia and hypoxia, low concentrations of **azo-PDT** only ablated the cell viability under hypoxia after irradiation, suggesting its hypoxia specificity (Fig. S5). The cytotoxicity of **azo-PDT** (2.5 μM) under normoxia or hypoxia, and with or without irridaition was summarized in Fig. 6 with **pyro** as a positive control. In contrast to **pyro** which shows photo-irradiation-dependent cytotoxicity either under normoxia or hypoxia, **azo-PDT** showed potent cytotoxicity only under hypoxia when photo-irradiated. This observation suggests that the cell ablation effect of **azo-PDT** relies on both photo-irradiation and hypoxia activation, which confirms the success of our designed probe. To make further confirmation that the hypoxia-photo-irradiation-dependent cytotoxicity of **azo-PDT** is indeed due to its induction of ROS generation, we checked the cellular ROS levels by staining cells with 2′, 7′-dichlorofluorescin diacetate, a ROS indicator. BEL-7402 cells were incubated with **azo-PDT** (2.5 μM) under normoxia or hypoxia for 6 h. Cells in the hypoxia group were then irradiated with LED light at 670 nm for 20 min, while cells in the normoxia group were kept under normal indoor light. Cells in all groups were then stained with 2′, 7′-dichlorofluorescin diacetate (5 μM) for 15 min, and then imaged under microscopy. As shown in Fig. S6, only cells in the hypoxia and irradiated group showed bright fluorescein fluorescence, indicating the upregulation of cellular ROS in this group. This result suggests that activated **azo-PDT** can still induce sufficient ROS even under hypoxia.

In summary, utilizing the resonant energy transfer between pyropheophorbide α and the quenched fluorophore **SiR-665**, we have developed a “pro-photosensitizer” that is activated under hypoxia in tumor cells. Due to the energy transfer between the photosensitizer and the quenched fluorophore, the pro-photosensitizer does not generate singlet oxygen to damage cells under normoxia. Under hypoxia, the azo group undergoes reductive cleavage, effectively separating the photosensitizer and fluorophore and, consequently disrupting the energy transfer between these groups and restoring the fluorescence of the fluorophore as well as the photoactivity of the photosensitizer. Applying this strategy, tumor-selective imaging and photodynamic therapy may be realized. We validated the feasibility of this strategy in live BEL–7402 cells. We have shown that 2.5 μM **azo-PDT** achieved real-time imaging and showed selective killing activity. Overall, **azo-PDT** may be a promising dual-functional imaging tool to detect cancer hypoxia as well as to achieve tumor-specific cancer therapy. Furthermore, the design strategy introduced in this study may be inspiring for future probe design.

## Methods

Detailed synthetic routes were described in supplementary information.

### Photophysical and photochemical property measurements

Deionized water was used to prepare all aqueous solutions. The concentration of phosphate buffer saline (PBS, pH 7.4) was 0.1 M. Dyes (**Pyro**, **SiR-665** or **azo-PDT**) were dissolved in dimethyl sulfoxide (DMSO) to make stock solutions. The UV absorption spectra were measured with a HITACHI U-3010 Spectrophotometer. The fluorescence measurements were obtained on an Agilent Cary Eclipse Fluorescence Spectrophotometer. The excitation and emission slit widths were 5 nm.

### Singlet oxygen detection by 1,3-diphenylisobenzofuran (DPBF)

Photosensitizers (**Pyro**, **azo-PDT** or methylene blue) with DPBF (40 μM) were dissolved in ethanol (EtOH). The solution was irradiated with a HollandStar GL-150 W LED light (670 nm). The rate of DPBF consumption (i.e., the slope) which corresponded to the relative efficiency of ^1^O_2_ generation by the irradiated photosensitizer was calculated according to the relative number of absorbed photons around the maximum absorbance of each dye^22^. Methylene blue (*Φ*(^1^O_2_) = 0.49 in EtOH) was used as a standard^23^. The ^1^O_2_ quantum yield was calculated by comparison with a standard photosensitizer, *Φ* = *Φ*_s_*k*I_*s*_/(*k*_s_*I)*,* where k and k_s_ were the rate constants for decomposition of DPBF by **Pyro** or **azo-PDT** and by methylene blue as a standard photosensitizer, respectively. I and I_s_ represented light absorbed by Pyro or azo-PDT and by methylene blue, respectively, which were determined by the wavelength of light source^20^.

### In vitro fluorescent assay with mouse liver microsomes

The enzyme assay in cuvette (hypoxic condition in vitro) was prepared by bubbling N_2_ into the 0.1 M PBS (pH 7.4) for at least 30 min. Mouse liver microsomes (226 µg/3 mL) from Research Institute for Liver Diseases (Shanghai) Co. Ltd and 5 µM **azo-PDT** was added into the reaction solution containing 5% DMF as a cosolvent. Then 50 µM NADPH as a cofactor for reductases was added.

### Cell lines and culture conditions

Human hepatoma cells BEL-7402 were supplied by collaborators. BEL-7402, KM12, A549 and DU145 cells were cultured in RPMI 1640 medium (Invitrogen) supplemented with 10% heat-inactivated FBS (Gibico). HepG2 and MCF7 cells were cultured in DMEM medium (Invitrogen) supplemented with 10% heat-inactivated FBS (Gibico). B16F10 cells were cultured in DMEM/F-12 (1/1) medium (Invitrogen) supplemented with 10% heat-inactivated FBS (Gibico). All cell lines were incubated at 37 ℃ under an atmosphere of 5% CO_2_ in air.

### Hypoxic condition for live cells fluorescence imaging

An O_2_ concentration of 1% was controlled by the Thermo Scientific Forma CO_2_ incubator by means of N_2_ substitution. Confocal Fluorescence images were obtained on Olympus IX83-FV3000. The fluorescence density was calculated using Image J software (NIH, Bethesda, MD, USA).

### Cell culture

#### Different cell lines

1.5 × 10^5^ cells were seeded onto 3.5 cm Petri dishes and cultured in corresponding medium with 10% (v/v) FBS and incubated for 12 h. The tested compounds were diluted to 5 μM, then the cells were incubated for 1 h under an atmosphere of 5% CO_2_ in air, thereafter the cells were incubated for 6 h under an atmosphere of 5% CO_2_ and 1% O_2_ (hypoxia) or for 6 h under an atmosphere of 5% CO_2_ in air (normoxia)**.** The medium was removed and washed with fresh medium**.** Confocal Fluorescence images were then obtained on Olympus IX83-FV3000.

#### Different hypoxia hours

1.5 × 10^5^ cells were seeded onto 3.5 cm Petri dishes and cultured in RPMI 1640 medium with 10% (v/v) FBS and incubated for 12 h. The tested compounds were diluted to 5 μM, then the cells were incubated for 1 h under an atmosphere of 5% CO_2_ in air, thereafter the cells were incubated for different hours under an atmosphere of 5% CO_2_ and 1% O_2_**.** The medium was removed and washed with fresh medium**.** Confocal Fluorescence images were then obtained on Olympus IX83-FV3000.

#### Different concentrations

1.5 × 10^5^ cells were seeded onto 3.5 cm Petri dishes and cultured in RPMI 1640 medium with 10% (v/v) FBS and incubated for 12 h. The tested compounds were diluted to different final concentrations, then the cells were incubated for 1 h under an atmosphere of 5% CO_2_ in air, thereafter the cells were incubated for 6 h under an atmosphere of 5% CO_2_ and 1% O_2_ (hypoxia) or for 6 h under an atmosphere of 5% CO_2_ in air (normoxia)**.** The medium wa**s** removed and washed with fresh medium. Confocal Fluorescence images were then obtained on Olympus IX83-FV3000.

### Hypoxia-triggered photodynamic ablation of tumor cells

#### Different concentrations

5 × 10^3^ cells were seeded on 96-well plates and cultured in RPMI 1640 medium with 10% (v/v) FBS for 24 h. The tested compounds were diluted to different final concentrations, then the cells were incubated for 1 h under an atmosphere of 5% CO_2_ in air, thereafter the cells were incubated for 6 h under an atmosphere of 5% CO_2_ and 1% O_2_ (hypoxia) or for 6 h under an atmosphere of 5% CO_2_ in air (normoxia)**.** Then the plate was irradiated by LED light (670 nm) for 20 min and cells were incubated under normal atmosphere for another 24 h. Cell viability was then measured using SRB assay or cck-8 assay.

## Supplementary information

Supplementary Information.
