# Supplementary material for: Microenvironment-triggered dual-activation of a photosensitizer- fluorophore conjugate for tumor specific imaging and photodynamic therapy
Source: Sci Rep. 2020 Jul 22;10:12127. doi: 10.1038/s41598-020-68847-w (PMC7376251; doi:10.1038/s41598-020-68847-w)
Supplement: Supplementary file 1 — Supplementary Information. [file 41598_2020_68847_MOESM1_ESM.docx]

**Supporting Information**

**Microenvironment-triggered dual-activation of a photosensitizer-fluorophore conjugate for tumor specific imaging and photodynamic therapy**

Chang Wang^a^, Shengdan Wang^a^, Yuan Wang^a^, Honghai Wu^a^, Kun Bao^b^, Rong Sheng*^a^, Xin Li*^a^

a. College of Pharmaceutical Sciences, Zhejiang University, Hangzhou, 310058, China

b. Collaborative Innovation Centre of Yangtze River Delta Region Green Pharmaceuticals, Zhejiang University of Technology, Hangzhou, 310014, China

* [Shengr@zju.edu.cn](mailto:Shengr@zju.edu.cn), [lixin81@zju.edu.cn](mailto:lixin81@zju.edu.cn)

**General information for chemistry experiments**

Anhydrous tetrahydrofuran (THF) was distilled from Na prior to use. Anhydrous N, N'-dimethylformamide (DMF) was distilled from CaH_2_. Other reagents were from commercial supplies and used without further purification. Reactions were monitored using thin layer chromatography (TLC), and confirmed by LC-MASS spectrometry.

^1^H and ^13^C NMR spectra were recorded on a Bruker Fourier transform 500 NMR spectrometer at room temperature. LC-mass spectra were measured with Shimadzu Prominence LC-20A using electrospray ionization (ESI). High-resolution mass data were taken on an Agilent 6224 TOF LC/MS spectrometer using electrospray ionization-time of flight (ESI-TOF). HPLC purification and analysis were performed on a reverse-phase column (Agilent 10 Prep-C18 250*21.2 mm and COSMOSIL Packed Column 5C_18_-AR-II 4.6ID*250 mm), the flow phases were eluent A (H_2_O containing 0.1% TFA(v/v)) and eluent B (CH_3_CN), fitted on an Agilent 1260 Infinity system.





**Scheme S1.** Synthetic route to **azo-PDT.** *a.* allyl bromide, K_2_CO_3_, KI, DMF, 80^o^C. *b.* POCl_3_, DMF, ClCH_2_CH_2_Cl, 0^o^C to reflux. *c.* NaBH_4_, THF, 0^o^C. *d*. 4-methylpent-3-en-2-one, I_2_/toluene, reflux. *e.* CH_3_I, Cs_2_CO_3_, DMF, 80^o^C. *f.* BF_3_·OEt_2_, CH_2_Cl_2_, reflux, *g.* i) n-BuLi, THF, -78^o^C, ii) SiCl_2_Me_2_, THF, -78^o^C to rt. *h.* KMnO_4_, acetone, -15^o^C. *i.* n-BuLi, THF, -78^o^C, ii) 2-bromotoluene, THF, -78^o^C to rt, iii) 1N HCl aq. *j.* i) NaBH_4_, MeOH; ii) methanesulfonic acid, Pd(PPh_3_)_4_, EtOH; iii) p-chloranil, CH_2_Cl_2_. *k.* 2-Oxazolidinone, 160^o^C. *l.* Di-tert-butyl pyrocarbonate (Boc_2_O), N,N-Diisopropylethylamine (DIPEA), 4-dimethylaminopyridine (DMAP), THF. *m.* NOBF_4_, CH_3_CN/Dimethyl sulfoxide (DMSO)=10/1, -15^o^C. *n.* HCl, CH_2_Cl_2_. *o.* [pyropheophorbide *a*](javascript:showMsgDetail('ProductSynonyms.aspx?CBNumber=CB91382063&postData3=CN&SYMBOL_Type=A');), N, N'-Diisopropylcarbodiimide (DIC), DMAP, DMF.





To a suspension of K_2_CO_3_ (48.0 g, 347 mmol) in DMF (200 ml) were added 3-bromoaniline (12.66 mL, 116 mmol) and allyl bromide (30.0 mL, 347 mmol), and the mixture was stirred at 80 °C for 14 h. After cooling, the reaction mixture was extracted with AcOEt. The organic layer was washed with brine, dried over Na_2_SO_4_ and evaporated to dryness. The residue was purified by column chromatography (silica gel, 1/40 AcOEt/Petroleum ether) to give pure 1 (24 g, 95.3 mmol, 82.2% yield).

**^1^H NMR (500 MHz, Chloroform-*d*):** δ 7.06 – 7.00 (m, 1H), 6.84 – 6.75 (m, 2H), 6.64 – 6.56 (m, 1H), 5.83 (ddt, *J* = 17, 10.5, 5 Hz, 2H), 5.23 – 5.11 (m, 4H), 3.90 (dt, *J* = 5, 2 Hz, 4H).

ESI-MS (m/z): [M+H]^+^ calcd. for C_12_H_15_BrN^+^: 252.0388; found 252.15.





DMF (26.4 ml, 341 mmol) in 1,2-dichloroethane (100 ml) was stirred at 0^o^C, then POCl_3_ (11.4 ml, 122 mmol) was added to the solution and stirring was continued for 15 minutes. Compound **1** (14.6 g, 58 mmol) in 1,2-dichloroethane (50 ml) was added, and the reaction mixture was refluxed for 3 hours. After cooling to room temperature, saturated Na_2_CO_3_ was added at 0^o^C, then the aqueous phase was extracted with CH_2_Cl_2_, and the combined organic phase was washed with water and brine, dried over Na_2_SO_4_, and evaporated to dryness. The residue was purified by column chromatography (silica gel, 1/20 AcOEt/Petroleum ether) to give pure 2 (14.4 g, 51.3 mmol, 88.4% yield).

**^1^H NMR (500 MHz, Chloroform-*d*):** δ 10.07 (d, *J* = 1 Hz, 1H), 7.77 (d, *J* = 9 Hz, 1H), 6.81 (d, *J* = 2.5 Hz, 1H), 6.64 (dd, *J* = 9, 2.5 Hz, 1H), 5.83 (ddt, *J* = 17, 10, 4.5 Hz, 2H), 5.23 (dq, *J* = 10, 1.5 Hz, 2H), 5.16 (dq, *J* = 17. 1.5 Hz, 2H), 3.98 (dt, *J* = 4.0, 2 Hz, 4H).

ESI-MS (m/z): [M+Na]^+^ calcd. for C_13_H_14_BrNONa^+^: 302.0156; found 302.10.





Compound **2** (11.2 g, 40 mmol) in THF (80 ml) was stirred at 0^o^C, then NaBH_4_ (3 g, 80 mmol) was slowly added to the solution and stirring was continued for 3h at room temperature. The reaction mixture was quenched with saturated NH_4_Cl, then the aqueous phase was extracted with AcOEt, and the combined organic phase was washed with water and brine, dried over Na_2_SO_4_, and evaporated to dryness. The residue was purified by column chromatography (silica gel, 1/10 AcOEt/Petroleum ether) to give pure 3 (10.4 g, 36.9 mmol, 92.3% yield).

**^1^H NMR (500 MHz, Chloroform-*d*):** δ 7.21 (d, *J* = 8.5 Hz, 1H), 6.87 (d, *J* = 2.5 Hz, 1H), 6.61 (dd, *J* = 8.5, 2.5 Hz, 1H), 5.82 (ddt, *J* = 17, 10.5, 4.5 Hz, 2H), 5.22 – 5.11 (m, 4H), 4.62 (s, 2H), 3.90 (dt, *J* = 4.5, 2 Hz, 4H), 1.89 (s, 1H).

ESI-MS (m/z): [M+Na]^+^ calcd. for C_13_H_16_BrNONa^+^: 304.0313; found 304.05.





A suspension of 3-bromoaniline (12.66 ml, 116 mmol), 4-methylpent-3-en-2-one (26.68 ml, 232 mmol), and iodine (3 g, 11.6 mmol) in toluene (200 ml) was refluxed for 48h. After cooling, the solvent was quenched with saturated Na_2_S_2_O_3_, then the aqueous phase was extracted with AcOEt, and the combined organic phase was washed with water and brine, dried over Na_2_SO_4_, and evaporated to dryness. The residue was purified by column chromatography (silica gel, 1/40 CH_2_Cl_2_/Petroleum ether) to give pure 2-1 (10.0 g, 39.7 mmol, 34.2% yield).

**^1^H NMR (500 MHz, Chloroform-*d*):** δ 6.89 (d, *J* = 8 Hz, 1H), 6.74 (dd, *J* = 8, 2 Hz, 1H), 6.61 (d, *J* = 2 Hz, 1H), 5.32 (d, *J* = 1.5 Hz, 1H), 1.95 (d, *J* = 1.5 Hz, 3H), 1.28 (s, 6H).

ESI-MS (m/z): [M+H]^+^ calcd. for C_12_H_15_BrN^+^: 252.0388; found 252.15.





Compound **2-1** (5.9g, 23.5 mmol), methyl iodide (5.7 ml, 94 mmol), and Caesium carbonate (4.6 g, 14.1 mmol) were dissolved in DMF (40 ml), and the mixture was refluxed for 4h. After cooling, the reaction mixture was quenched by adding saturated NH_4_Cl. Then the aqueous phase was extracted with AcOEt, and the combined organic phase was washed with water and brine, dried over Na_2_SO_4_, and evaporated to dryness. The residue was purified by column chromatography (silica gel, 1/40 AcOEt/Petroleum ether) to give pure 2-2 (5.5 g, 20.7 mmol, 87.9% yield).

**^1^H NMR (500 MHz, Chloroform-*d*):** δ 6.87 (d, *J* = 8.0 Hz, 1H), 6.75 (d, *J* = 8.0 Hz, 1H), 6.62 (s, 1H), 5.29 (q, *J* = 1.5 Hz, 1H), 2.77 (s, 3H), 1.95 (d, *J* = 1.5 Hz, 3H), 1.30 (s, 6H).

ESI-MS (m/z): [M+H]^+^ calcd. for C_13_H_17_BrN^+^: 266.0544; found 266.15.





Compound **3** (1.36 g, 4.8 mmol ) and compound **2-2** (1.28 g, 4.8 mmol ) were dissolved in CH_2_Cl_2_ (10 ml). BF_3_·OEt_2_ complex (1.3 ml, 9.6 mmol ) was added to the solution at 0^o^C. The reaction mixture was refluxed overnight, then cooled to room temperature, and saturated NaHCO_3_ was added to it. The aqueous phase was extracted with CH_2_Cl_2_. The combined organic phase was washed with water and brine, dried over Na_2_SO_4_, and evaporated to dryness. The residue was purified by column chromatography (silica gel, 1/100 AcOEt/Petroleum ether) to give pure 4 (2.25 g, 4.24 mmol, 88.3% yield) as a colorless oil.

**^1^H NMR (500 MHz, Chloroform-*d*):** δ 6.91 (s, 1H), 6.78 (s, 1H), 6.75 (d, *J* = 8.5 Hz, 1H), 6.71 (s, 1H), 6.53 (s, 1H), 5.86 – 5.78 (m, 2H), 5.29 (s, 1H), 5.18 – 5.13 (m, 4H), 3.96 (s, 2H), 3.87 (dt, *J* = 5, 2 Hz, 4H), 2.78 (s, 3H), 1.86 (d, *J* = 1.5 Hz, 3H), 1.30 (s, 6H).

ESI-HRMS (m/z): [M+H]^+^ calcd. for C_26_H_31_Br_2_N_2_^+^: 531.0828; found 531.0831.





Compound **4** (800 mg, 1.51 mmol ) was dissolved in anhydrous THF (10 ml) and the solution was stirred at -78^o^C. *n*-Butyl lithium cyclohexane/*n*-hexane solution (2.5 M, 1.8 ml, 4.53 mmol ) was added slowly to the reaction mixture, and stirring was continued for 1h. Dichlorodimethylsilane (0.4 ml, 3.02 mmol ) was added dropwise via a syringe at -78^o^C. The reaction mixture was warmed up to room temperature, stirred overnight, quenched with saturated NH_4_Cl. The aqueous phase was extracted with CH_2_Cl_2_. The combined organic phase was washed with water and brine, dried over Na_2_SO_4_, and evaporated to dryness. The resultant oil was dissolved in acetone, and the solution was stirred at -15^o^C. KMnO_4_ (359 mg, 2.27 mmol ) was added portionwise to it, and stirring was continued for 4 hours, then diluted with CH_2_Cl_2_. The solution was filtered through a Celite pad, and the filtrate was evaporated. The residue was purified by column chromatography (silica gel, 1/20 AcOEt/Petroleum ether) to give pure 6 (74 mg, 0.17 mmol, 11% yield) as a yellow solid.

**^1^H NMR (****500 MHz, Chloroform-*d*):** δ 8.35 (d, *J* = 8.9 Hz, 1H), 8.17 (s, 1H), 6.83 (dd, *J* = 9, 3 Hz, 1H), 6.80 (d, *J* = 3 Hz, 1H), 6.57 (s, 1H), 5.88 (ddt, *J* = 17.5, 10, 5 Hz, 2H), 5.32 (d, *J* = 1.5 Hz, 1H), 5.23 (q, *J* = 1.5 Hz, 2H), 5.20 (dq, *J* = 5, 1.5 Hz, 2H), 4.03 (dt, *J* = 5, 2 Hz, 4H), 2.94 (s, 3H), 2.07 (d, *J* = 1.5 Hz, 3H), 1.38 (s, 6H), 0.43 (s, 6H).

**^13^C NMR (126 MHz, Chloroform-*d*):** δ 185.23, 150.16, 147.00, 140.87, 140.50, 133.13, 131.66, 130.09, 129.97, 129.75, 128.15, 124.98, 123.45, 116.66, 114.84, 113.48, 112.59, 57.32, 52.79, 31.16, 28.74, 18.88, -1.00.

ESI-HRMS (m/z): [M+H]^+^ calcd. for C_28_H_35_N_2_Si^+^: 443.2519; found 443.2515.





2-bromotoluene (0.75 ml, 6.24 mmol ) was dissolved in anhydrous THF (10 ml) and the solution was stirred at -78^o^C. *n*-Butyl lithium cyclohexane/*n*-hexane solution (2.5 M, 1.8 ml, 4.53 mmol ) was added slowly to the reaction mixture, and stirring was continued for 1h. Compound **6** (700 mg, 1.58 mmol ) was added dropwise via a syringe at -78^o^C. The reaction mixture was warmed up to room temperature, stirred for 2h, quenched with 1 N HCl. The aqueous phase was extracted with CH_2_Cl_2_. The combined organic phase was washed with water and brine, dried over Na_2_SO_4_, and evaporated to dryness. The residue was purified by column chromatography (silica gel, 1/20 CH_2_Cl_2_/MeOH ) to give pure 7 (590 mg, 1.14 mmol, 72.2% yield) as a dark green solid.

**^1^H NMR (500 MHz, Chloroform-*d*):** δ 7.44 (td, *J* = 7.5, 1.5 Hz, 1H), 7.36 (d, *J* = 12 Hz, 3H), 7.14 (d, *J* = 3 Hz, 1H), 7.12 – 7.07 (m, 1H), 7.05 (d, *J* = 9.5 Hz, 1H), 6.71 (s, 1H), 6.60 (dd, *J* = 9.5,3 Hz, 1H), 5.89 (ddt, *J* = 17, 10, 4.5 Hz, 2H), 5.43 (s, 1H), 5.34 – 5.28 (m, 2H), 5.23 (d, *J* = 17 Hz, 2H), 4.19 (d, *J* = 4.5 Hz, 4H), 3.53 (s, 3H), 2.08 (s, 3H), 1.57 – 1.51 (m, 9H), 0.67 (s, 3H), 0.66 (s, 3H).

**^13^C NMR (126 MHz, Chloroform-*d*):** δ 169.67, 153.74, 153.20, 152.52, 147.83, 141.41, 139.53, 136.56, 133.68, 132.00, 131.82, 131.22, 129.98, 129.83, 129.15, 129.11, 126.77, 126.71, 124.23, 122.05, 121.47, 119.02, 114.90, 62.11, 54.55, 35.28, 30.29, 30.24, 20.57, 18.56, 0.30, 0.00.

ESI-HRMS (m/z): [M]^+^ calcd. for C_35_H_41_N_2_Si^+^: 517.3034; found 517.3036.





To Pd(PPh_3_)_4_ (958 mg, 0.86 mmol) under N_2_ was added compound 7 (960 mg, 1.86 mmol) and methanesulfonic acid (0.6 ml, 9.3 mmol) in EtOH (10 ml) . The homogeneous solution was heated to reflux under N_2_ for 48 h, then cooled to room temperature, and NaBH_4_ was added to the solution at room temperature until the intense blue color disappeared. The reaction mixture was quenched with water, then the solution was filtered through a Celite pad, and the filtrate was evaporated. The residue was purified by column chromatography (silica gel, 1/20 AcOEt/Petroleum ether) . The resultant solid was dissolved in CH_2_Cl_2_ (10 ml), p-Chloranil (549 mg, 2.23 mmol ) was added, and the whole was stirred for 1h. The solution was filtered through a Celite pad, and the filtrate was evaporated. The residue was purified by column chromatography (silica gel, 1/15 CH_2_Cl_2_/MeOH ) to give pure SiR-665 (407 mg, 0.93 mmol, 50% yield) as a dark blue solid.

**^1^H NMR (500 MHz, DMSO-*d*_6_):** δ 8.32 (s, 2H), 7.46 – 7.30 (m, 3H), 7.27 (s, 1H), 7.19 (s, 1H), 7.10 (d, *J* = 7.5 Hz, 1H), 6.88 (d, *J* = 9.5 Hz, 1H), 6.60 (d, *J* = 9.5 Hz, 1H), 6.49 (s, 1H), 5.55 (s, 1H), 3.25 (s, 3H), 1.95 (s, 3H), 1.40 (d, *J* = 8.0 Hz, 9H), 0.52 (s, 3H), 0.49 (s, 3H).

**^13^C NMR (126 MHz, Chloroform-*d*):** δ 168.29, 157.75, 149.38, 149.23, 149.00, 142.98, 138.86, 135.67, 131.77, 130.12, 129.92, 128.92, 128.79, 127.79, 127.63, 126.31, 125.61, 122.51, 118.03, 117.58, 59.65, 32.50, 29.77, 29.38, 19.51, 17.68, -0.93, -1.16.

ESI-HRMS (m/z): [M]^+^ calcd. for C_29_H_33_N_2_Si^+^: 437.2408; found 437.2411.





The compound **3-1** was synthesized according to the reference^1^ and without further purifying.





All of the compound **3-1** dissolved in THF (50 ml) was added DIPEA (19 ml). Then Boc_2_O (17 ml, 74mmol) and DMAP (451mg, 3.69 mmol) was added to the reaction mixture, and stirring was continued overnight. The aqueous phase was extracted with CH_2_Cl_2_. The combined organic phase was washed with water and brine, dried over Na_2_SO_4_, and evaporated to dryness. The residue was purified by column chromatography (silica gel, 1/20 AcOEt/Petroleum ether) to give pure 3-2 (2 g, 8.14 mmol, 22% two-step total yield) as a white solid.

**^1^H NMR (500 MHz, Chloroform-*d*):** δ 7.24 (t, *J* = 7.5 Hz, 2H), 6.80 – 6.68 (m, 3H), 3.45 (t, *J* = 6.5 Hz, 2H), 3.32 (q, *J* = 6.5 Hz, 2H), 2.95 (s, 3H), 1.45 (d, *J* = 4.5 Hz, 9H).

ESI-HRMS (m/z): [M+H]^+^ calcd. for C_14_H_23_N_2_O_2_^+^: 251.1760; found 251.1756.





**SiR-665** (266 mg, 0.61 mmol) was dissolved in a mixture of CH3CN/DMSO (10/1 v/v, 10 ml) at -15°C. Solid NOBF_4_ salt was added by portions (108 mg, 0.92 mmol) and the resulting reaction mixture was stirred vigourously for 30 min. Thereafter, compound **3-2** (384 mg, 1.53 mmol) was slowly added to the pre-formed *N*-nitrosamine/diazonium salt intermediate and the resulting reaction mixture was stirred for further 30 min. The aqueous phase was extracted with CH_2_Cl_2_. The combined organic phase was washed with water and brine, dried over Na_2_SO_4_, and evaporated to dryness. The residue was purified by column chromatography (silica gel, 1/1 AcOEt/Petroleum ether to 1/20 CH_2_Cl_2_/MeOH ) to give pure 9 (150 mg, 0.21 mmol, 35.2% yield) as a dark blue solid.

**^1^H NMR (500 MHz, DMSO-*d*_6_):** δ 8.28 (s, 1H), 7.81 (d, *J* = 9 Hz, 2H), 7.73 (d, *J* = 9 Hz, 2H), 7.56 – 7.48 (m, 2H), 7.45 (s, 1H), 7.24 (d, *J* = 7.5 Hz, 1H), 7.14 (d, *J* = 9 Hz, 1H), 6.96 (s, 1H), 6.89 (d, *J* = 9 Hz, 2H), 6.68 (s, 1H), 5.94 (s, 1H), 3.65 (s, 3H), 3.54 (s, 2H), 3.16 (s, 2H), 3.07 (s, 3H), 2.02 (s, 3H), 1.59 – 1.46 (m, 9H), 1.34 (s, 9H), 0.69 (s, 3H), 0.66 (s, 3H).

ESI-HRMS (m/z): [M]^+^ calcd. for C_43_H_52_N_5_O_2_Si^+^: 698.3885; found 698.3885.





Compound **9 (**84 mg, 0.12 mmol**)** was dissolved in CH_2_Cl_2_ (9 ml) under N_2_. Saturated HCl in AcOEt (3 ml) was slowly added and the resulting reaction mixture was stirred vigourously for 1h. TLC detected the reaction termination, then the resulting reaction mixture was evaporated to dryness to give compound **10** without further purifying.

[Pyropheophorbide-α](javascript:showMsgDetail('ProductSynonyms.aspx?CBNumber=CB91382063&postData3=CN&SYMBOL_Type=A');) (50 mg, 0.09 mmol) was dissolved in anhydrous DMF (10 ml) under N_2_ at room temperature. DIC (28μl, 0.18mmol) in anhydrous DMF (0.1 ml) and HOBt (24 mg, 0.18mmol) in anhydrous DMF (0.1 ml) were added and the resulting reaction mixture was stirred vigourously for 6h. Thereafter, all of compound **10** (72 mg, 0.12 mmol) in DMF (5 ml) was slowly added to and the resulting reaction mixture was stirred for further 30 min. The aqueous phase was extracted with CH_2_Cl_2_. The combined organic phase was washed with water and brine, dried over Na_2_SO_4_, and evaporated to dryness. The residue was purified by preparative HPLC (eluent; 0.1% CF_3_COOH H_2_O/MeCN =10/90 to 1/99, 30 min) to give pure azo-PDT (30 mg, 0.03 mmol, 33.3% yield) as a dark green solid.

**^1^H NMR (500 MHz, DMSO-*d*_6_):** δ 9.64 (s, 1H), 9.40 (s, 1H), 8.84 (s, 1H), 8.23 (s, 1H), 8.19 (dd, *J* = 17.8, 11.5 Hz, 1H), 7.82 (s, 1H), 7.74 (d, *J* = 8.5 Hz, 2H), 7.71 (s, 1H), 7.67 (d, *J* = 8.5 Hz, 1H), 7.50 (dt, *J* = 14.5, 7.5 Hz, 2H), 7.43 (t, *J* = 7.5 Hz, 1H), 7.22 (d, *J* = 7.5 Hz, 1H), 7.10 (d, *J* = 9.5 Hz, 1H), 6.73 (d, *J* = 9.0 Hz, 2H), 6.65 (s, 1H), 6.35 (dd, *J* = 18, 1.5 Hz, 1H), 6.18 (dd, *J* = 11.5, 1.5 Hz, 1H), 5.92 (s, 1H), 5.29 (s, 1H), 5.20 (d, *J* = 20 Hz, 1H), 5.07 (d, *J* = 20 Hz, 1H), 4.48 (d, *J* = 8 Hz, 1H), 4.25 (d, *J* =8 Hz, 1H), 3.64 (d, *J* = 14.5 Hz, 5H), 3.53 (s, 3H), 3.40 (s, 4H), 3.18 (s, 3H), 3.12 – 3.04 (m, 3H), 2.85 (s, 3H), 2.78 (dd, *J* = 25, 14 Hz, 1H), 2.22 (td, *J* = 19.5, 17, 8.5 Hz, 2H), 2.00 (s, 3H), 1.96 (d, *J* = 11 Hz, 2H), 1.73 (d, *J* = 7.5 Hz, 3H), 1.59 (t, *J* = 7.5 Hz, 3H), 1.52 (d, *J* = 3.5 Hz, 6H), 0.67 – 0.56 (m, 6H), -2.02 (s, 1H).

**^13^C NMR (126 MHz, DMSO-*d_6_*):** δ 195.34, 172.30, 172.11, 162.29, 161.50, 158.90, 158.61, 158.33, 158.04, 154.31, 153.94, 153.27, 152.90, 152.69, 149.86, 147.97, 144.72, 143.37, 141.18, 140.72, 139.67, 138.03, 137.20, 135.97, 135.60, 135.28, 135.04, 133.92, 131.72, 131.39, 130.98, 130.37, 130.18, 129.14, 129.07, 128.06, 126.07, 125.04, 124.21, 122.88, 119.31, 116.98, 114.66, 112.34, 111.62, 106.26, 104.13, 96.50, 93.80, 63.29, 51.25, 50.52, 49.43, 47.60, 40.43, 38.24, 35.46, 32.10, 29.73, 27.50, 27.37, 22.91, 19.06, 18.63, 17.46, 16.61, 12.02, 11.62, 10.85, -1.42, -1.79.

ESI-HRMS (m/z): [M]^+^ calcd. for C_71_H_76_N_9_O_2_Si^+^: 1114.5886; found 1114.5886.


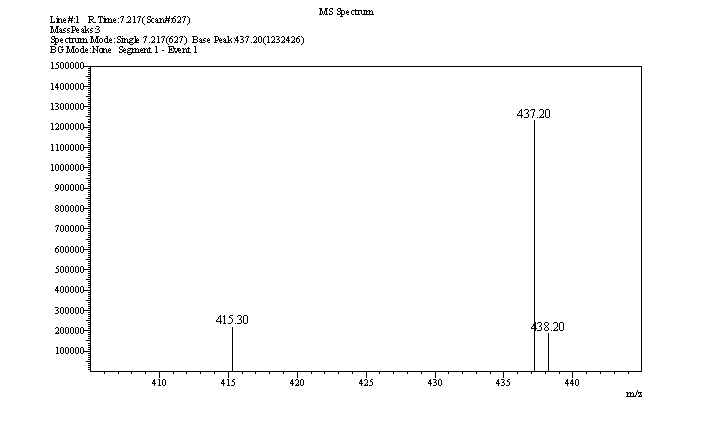


**Fig. S1** MS spectra of the fluorescent product of **azo-PDT** upon reduction by NaHS in PBS solution. The peak *m/z* 437.2 ([M]^+^) indicated its transformation to the amino**-SiR-665.**

**
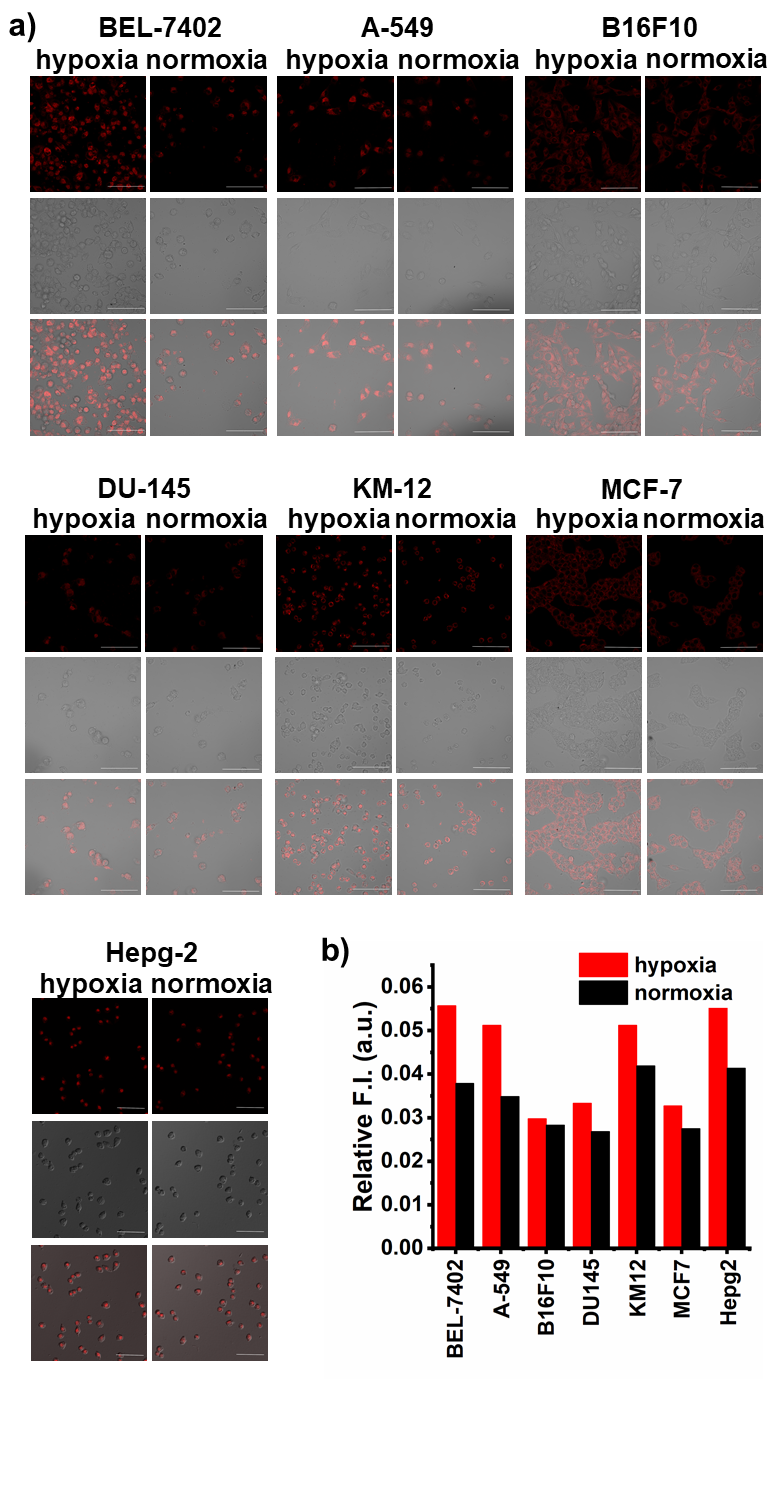
**

**Fig. S2** a) Fluorescence confocal microscopy images of **azo-PDT** in BEL-7402, A-549, B16F10, DU-145, KM-12, MCF-7 and Hepg-2 cells. Cells were incubated with **azo-PDT** (5 μM) containing DMSO (0.1%) and ethoxylated hydrogenated castor oil (RH40) (0.1%) as cosolvents under normoxia for 1 h, then transferred to hypoxia or normoxia for 6 h. Scale bar: 100 μm. The excitation and emission wavelengths were 640 and 650-750 nm, respectively; b) The relative fluorescence intensity calculated by Image J.


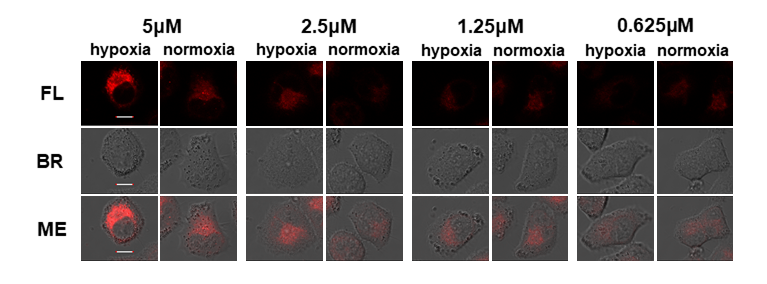


**Fig. S3** Concentration-dependent change of the fluorescence intensity from BEL-7402 cells after 6 h incubation containing DMSO (0.1%) and RH40 (0.1%) as cosolvent. Scale bar: 10 μm. The excitation and emission wavelengths were 640 and 650-750 nm, respectively.


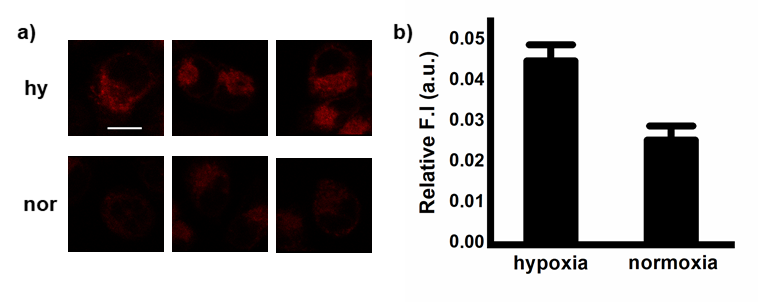


**Fig. S4** BEL-7402 cells were incubated with 2.5 μM **azo-PDT** for 6h under hypoxia or normoxia and then were imaged with λex 640 nm and λem 650-750 nm. Scale bar: 10 μm. The relative intracellular fluorescence intensities were calculated by Image J. Error bar represented S.D (n=3).





**Fig. S5** Survival viabilities of BEL-7402 cells treated by **azo-PDT** and **pyro** at different concentrations incubating under normoxia or hypoxia, further treatment with or without photoirradiation, using the SRB assay to monitor cell viabilities. a) incubating under normoxia, further treatment without photoirradiation; b) incubating under hypoxia, further treatment without photoirradiation; c) incubating under normoxia, further treatment with photoirradiation (20 min); and d) incubating under hypoxia, further treatment with photoirradiation (20 min). The power of LED lamp was about 150 W and the distance of irradiation was 6.5 cm.


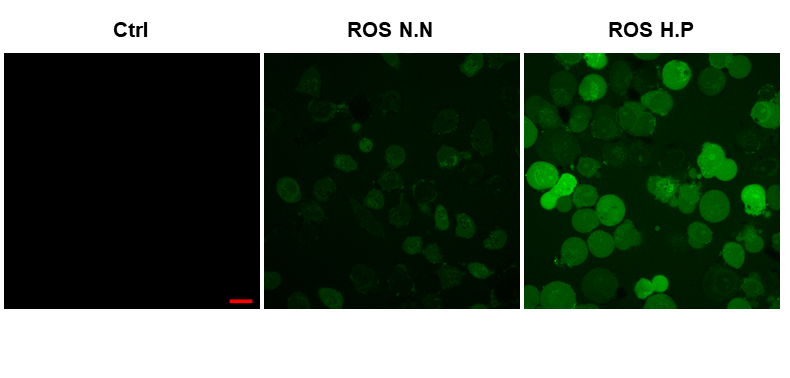


**Fig. S6** Imaging **azo-PDT**-induced ROS in BEL-7402 cells using 5 μM 2′, 7′-dichlorofluorescin diacetate as a ROS indicator. **Ctrl:** cells incubated under normoxia without photo-irradiation under 670 nm were stained with 5 μM ROS indicator; **ROS N.N**: cells were first incubated with 2.5 μM **azo-PDT** under normoxia in the dark for 6 h, and then stained with 5 μM ROS indicator, and then imaged; **ROS H.P**: cells were first incubated with 2.5 μM **azo-PDT** under hypoxia for 6 h, exposed to 670 nm light for 20 min, stained with 5 μM ROS indicator, and then imaged. The power of LED lamp was about 150 W and the distance of irradiation was 6.5 cm. Scale bar: 20 μm. The excitation and emission wavelengths were 488 and 493-543 nm, respectively.

**Reference**

1. Poindexter, G. S., Owens, D. A., Dolan, P. L., & Woo, E. The use of 2-oxazolidinones as latent aziridine equivalents. 2. Aminoethylation of aromatic amines, phenols, and thiophenols. *The Journal of Organic Chemistry* **57,** 6257-6265 (1992).

**NMR and MS spectra**

**^1^H NMR (500 MHz, CDCl_3_)**

**
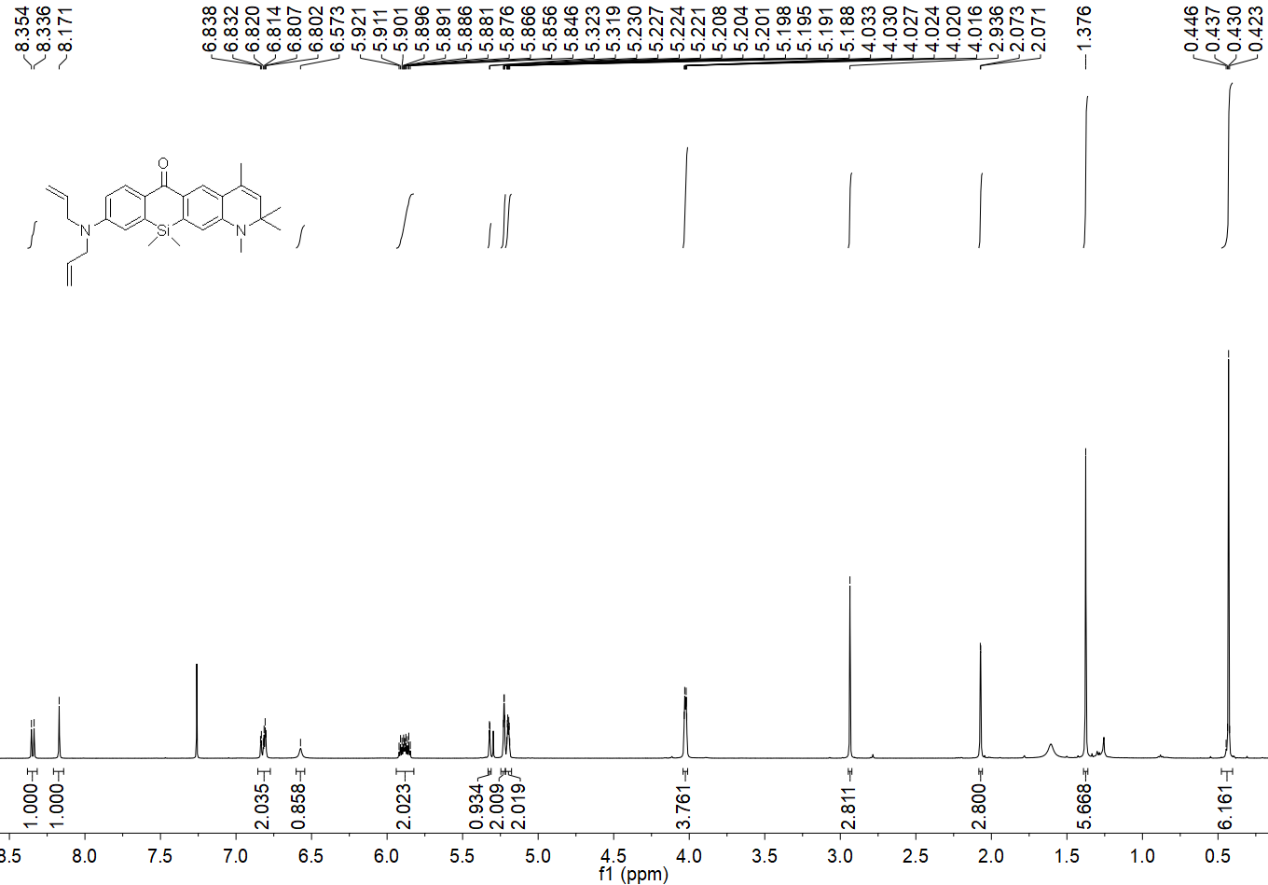
**


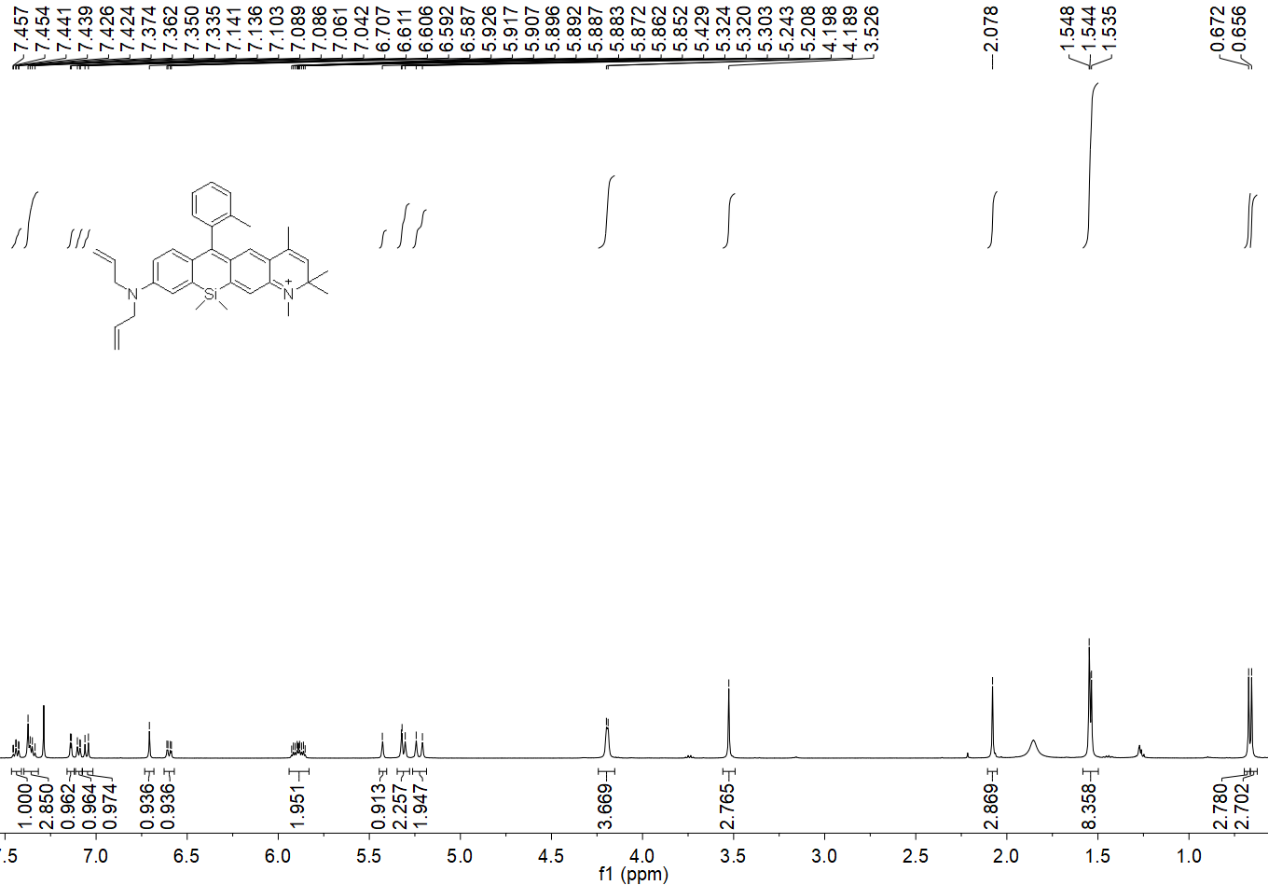


**^1^H NMR (500 MHz, DMSO-d6)**


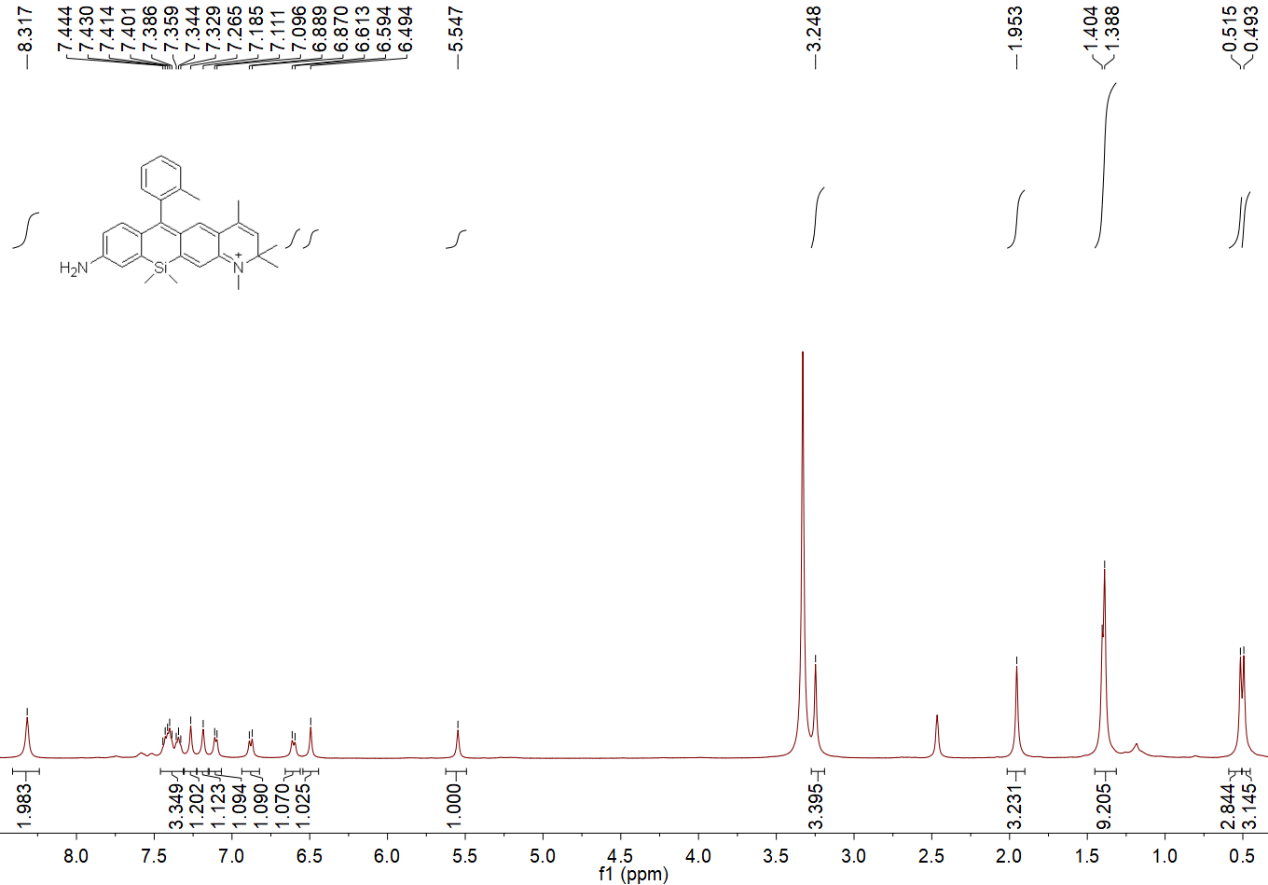


**
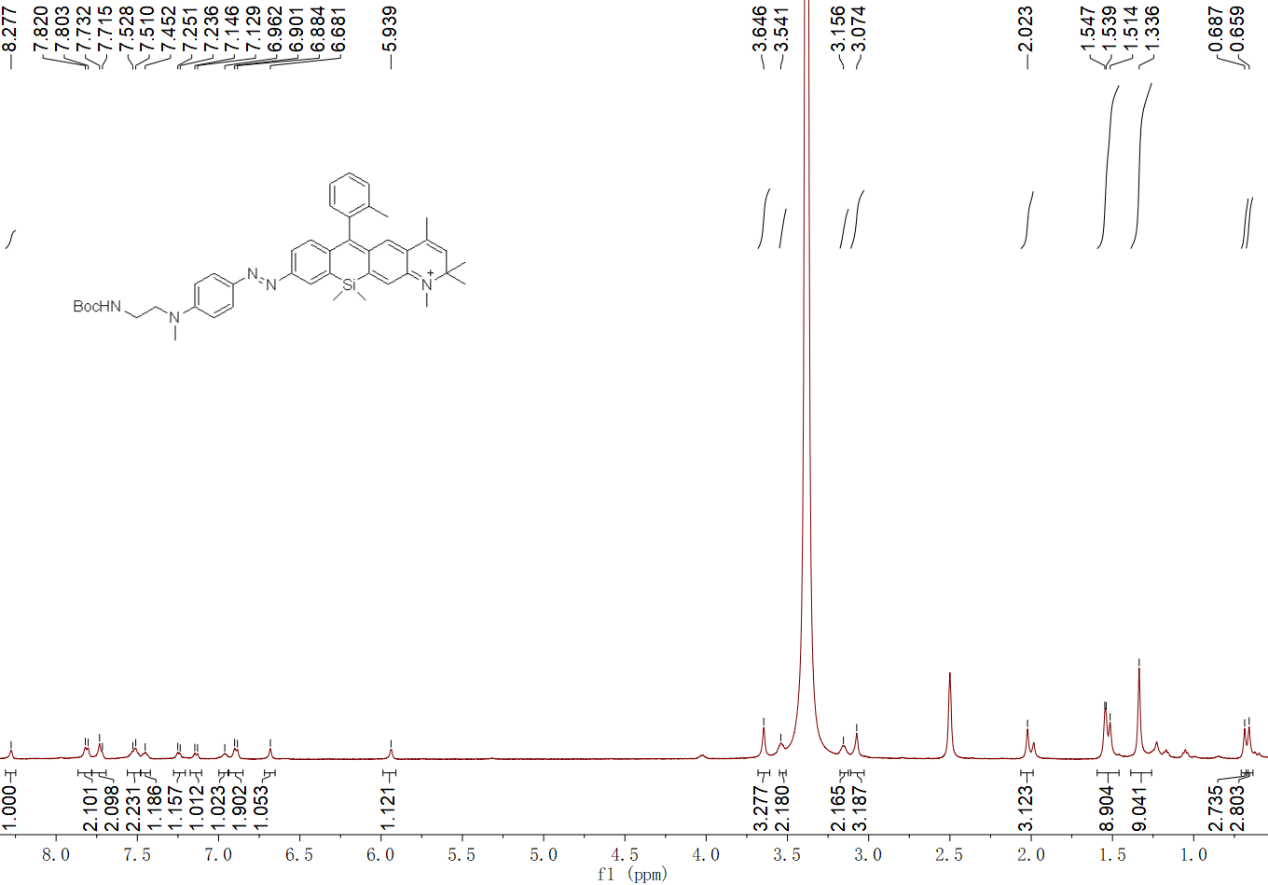
**


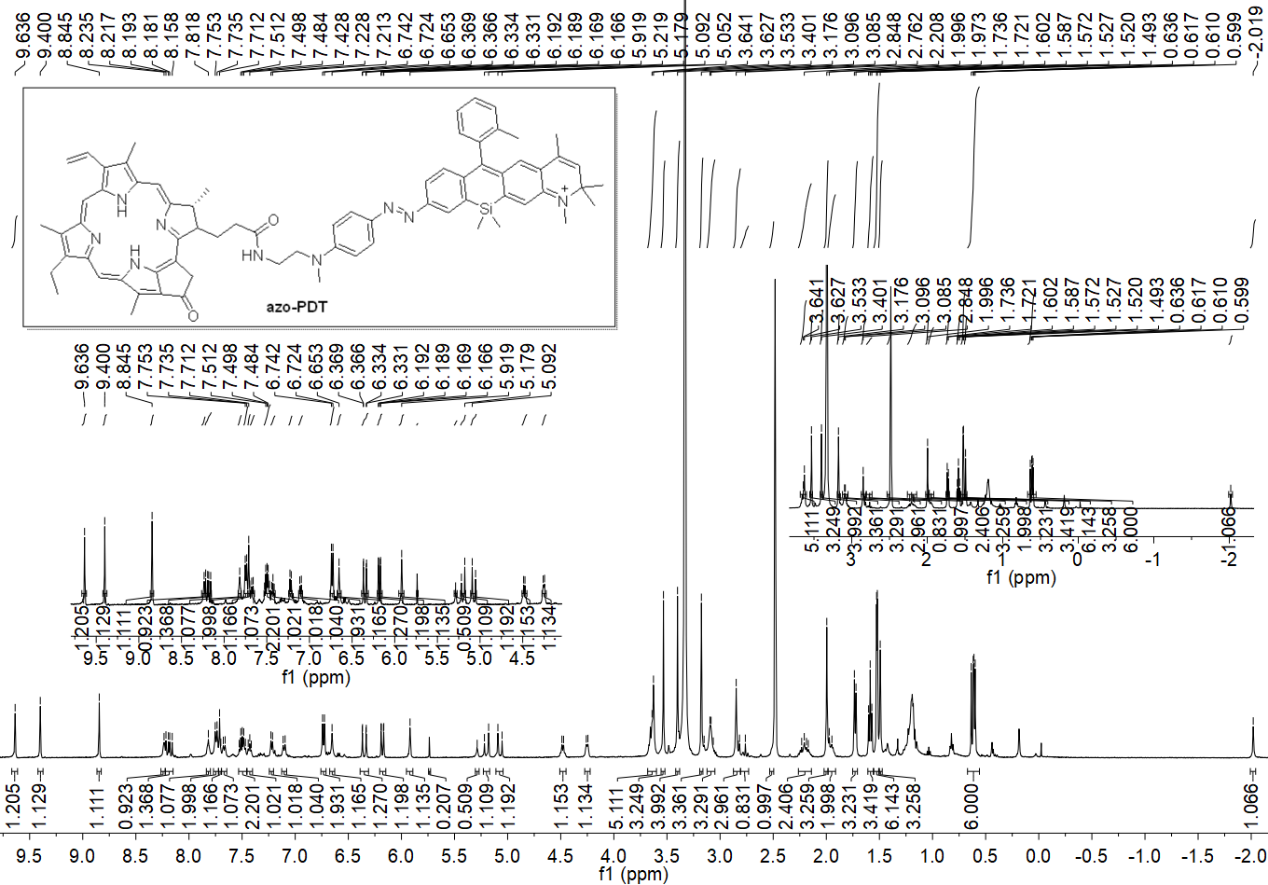


**^13^C NMR (126 MHz, CDCl_3_)**

**
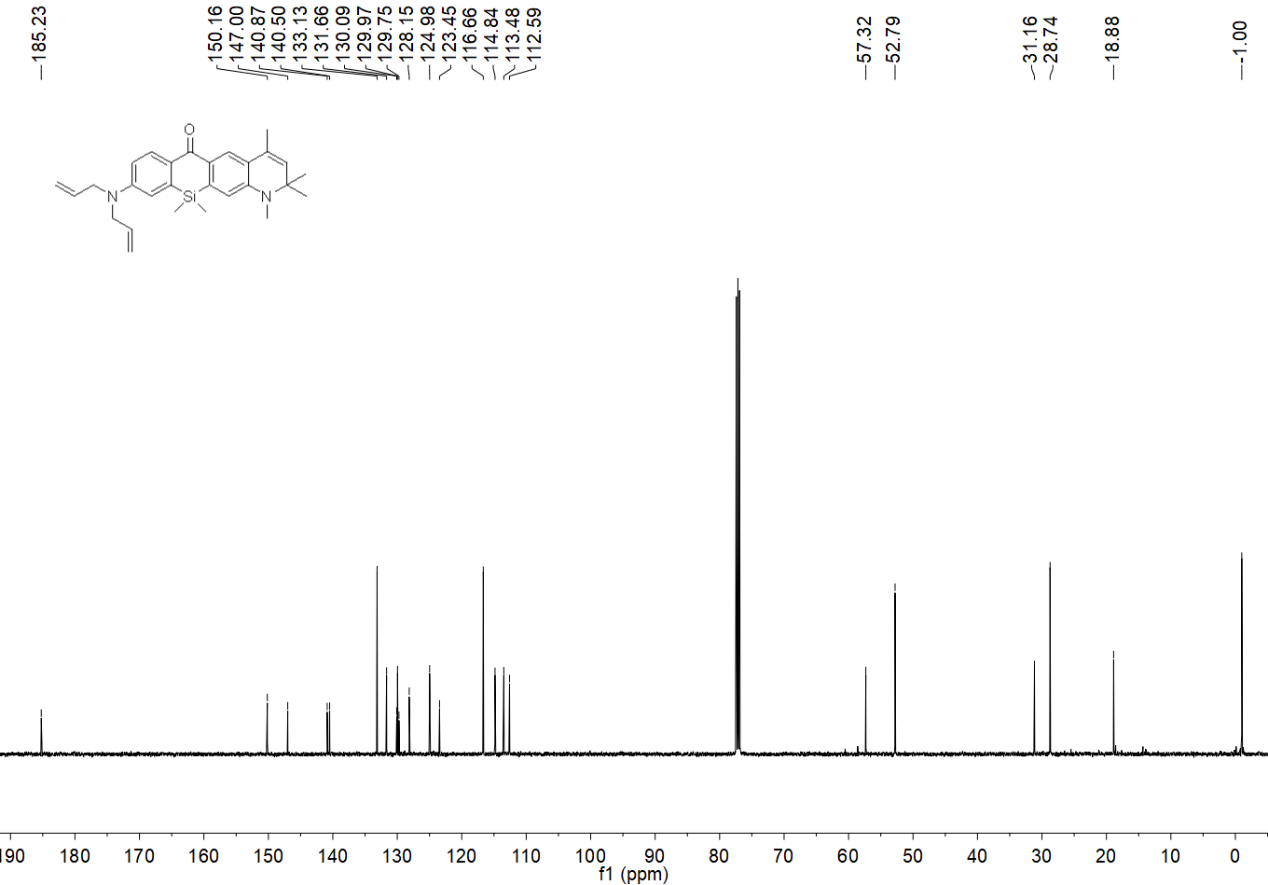
**


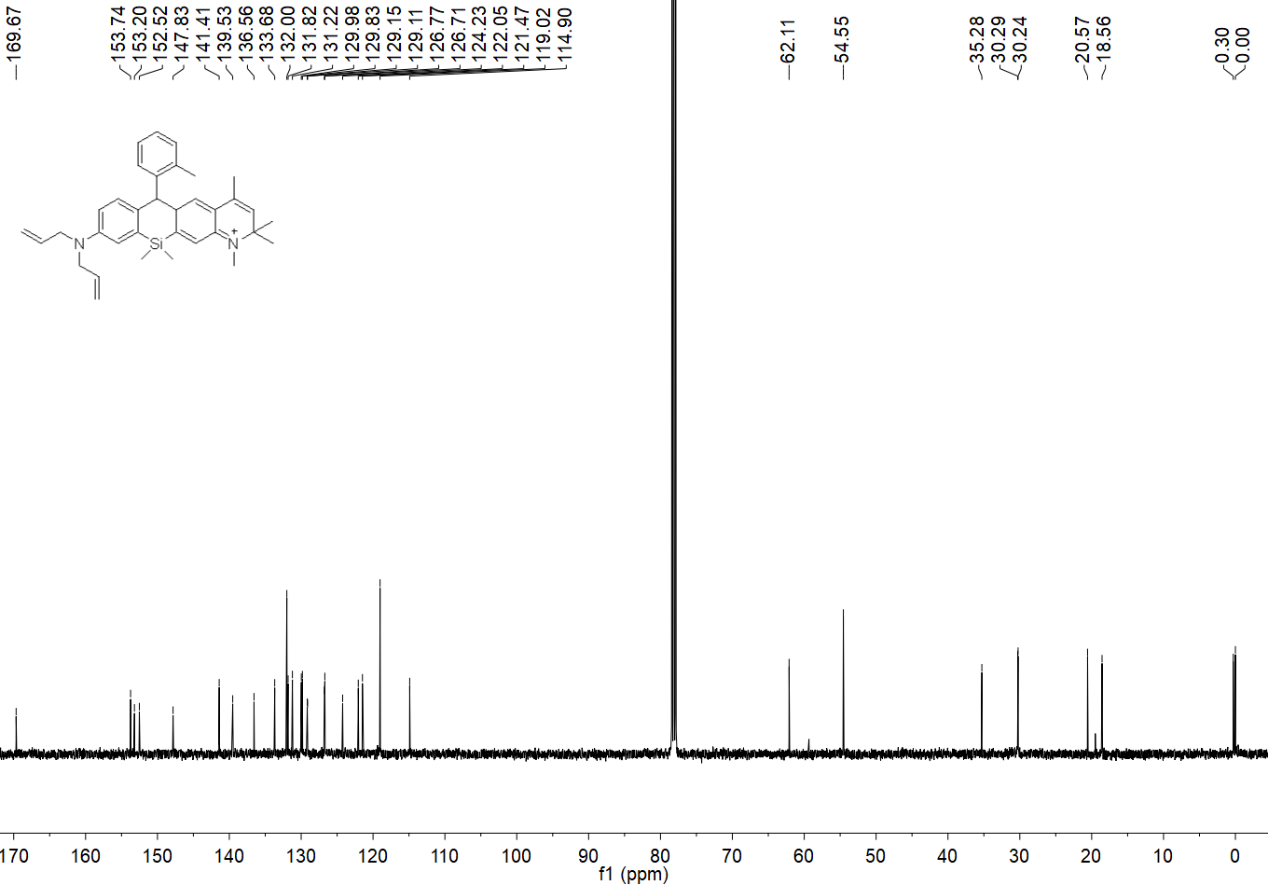


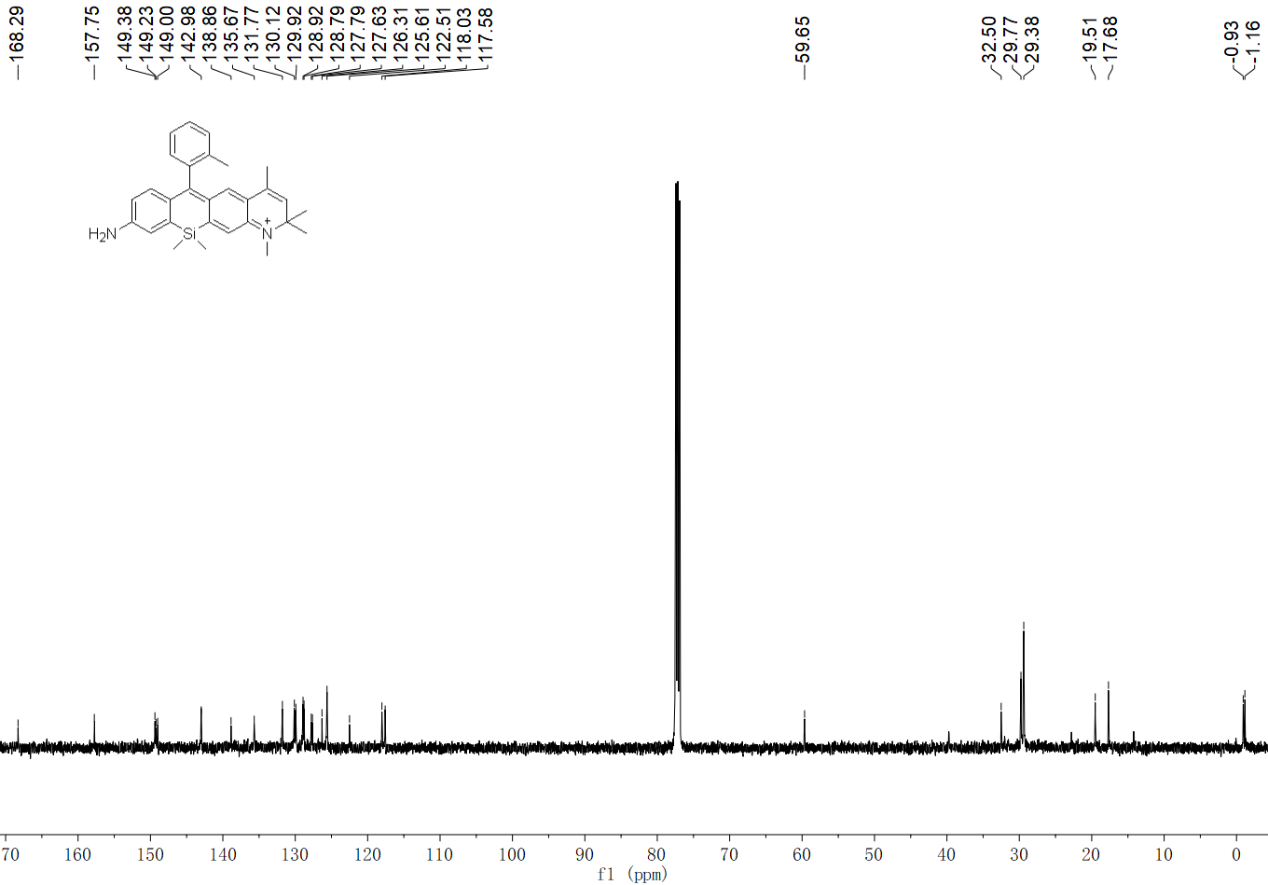


**^13^C NMR (126 MHz, DMSO-d6)**


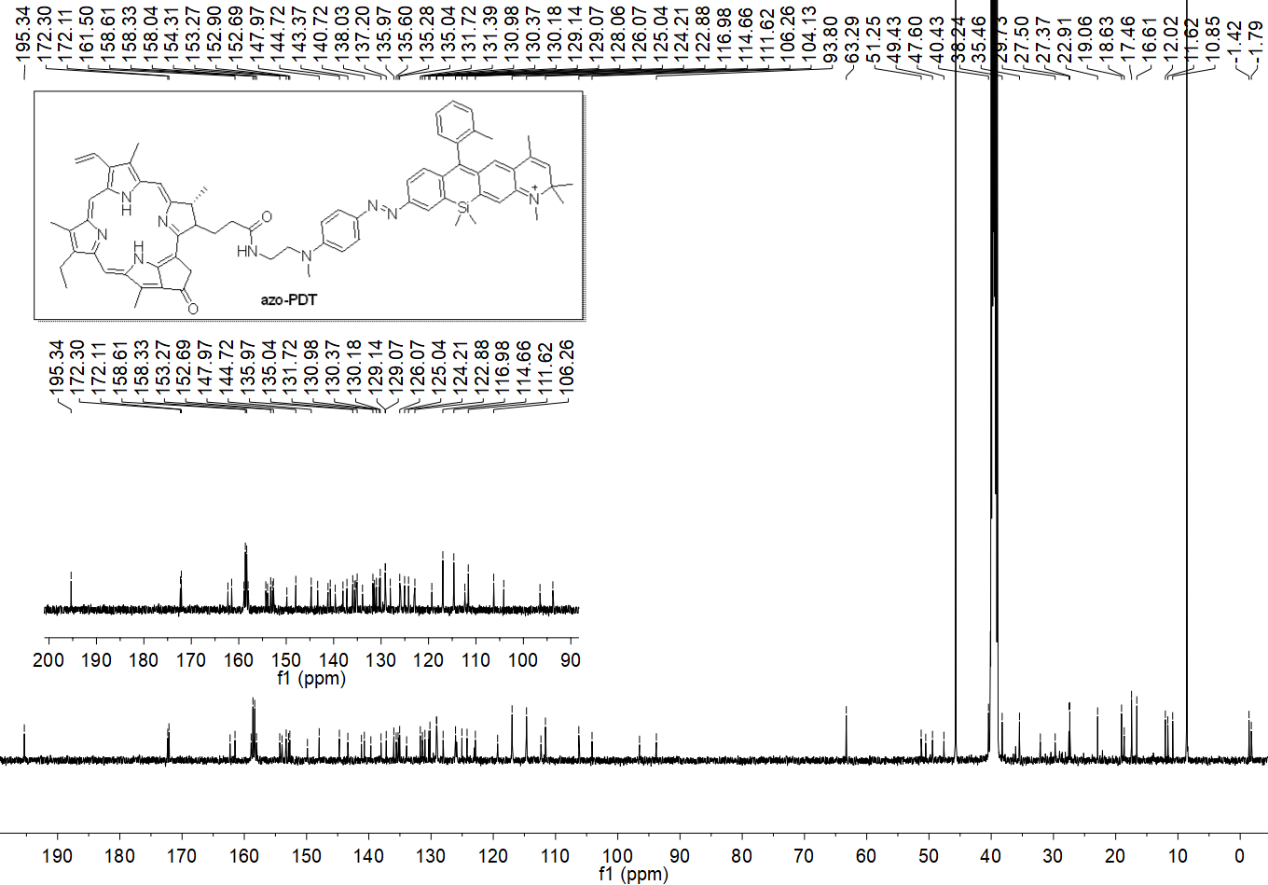


**ESI-HRMS**





**HPLC trace of azo-PDT**


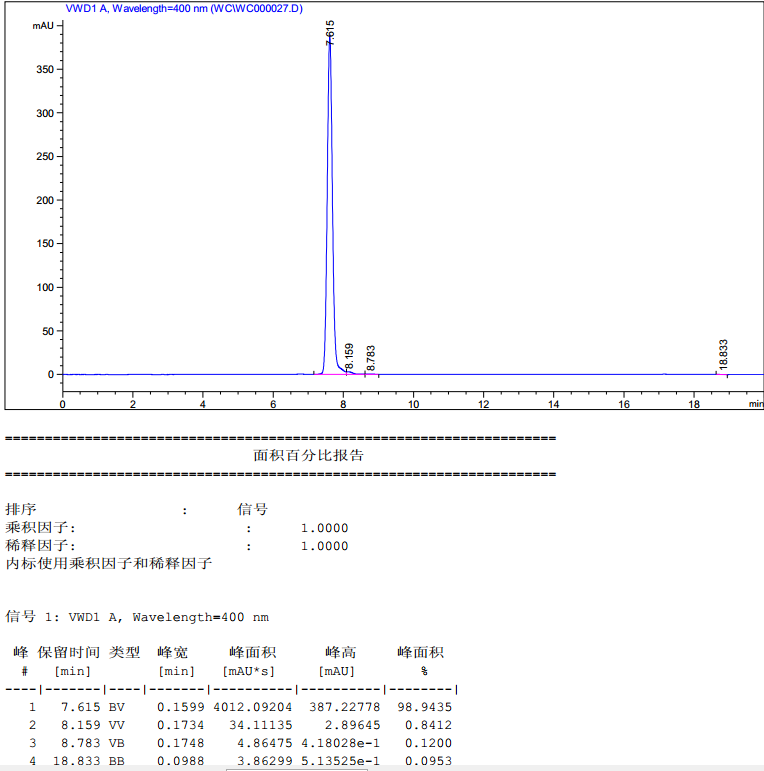


Column: Agilent 10 Prep-C18 250*21.2 mm; Eluent: 0.1% CF_3_COOH, H_2_O/MeCN =10/90, 2 min; 0.1% CF_3_COOH, H_2_O/MeCN =10/90 to 1/99, 8 min; 0.1% CF_3_COOH, H_2_O/MeCN =1/99, 10min.
